# Synthesis of Quillaic Acid through Sustainable C–H Bond Activations

**DOI:** 10.1021/acs.joc.3c02958

**Published:** 2024-04-10

**Authors:** Yi-Chi Wang, Cheng-Ru Chen, Chien-Yi Chen, Pi-Hui Liang

**Affiliations:** School of Pharmacy, College of Medicine, National Taiwan University, Taipei 100, Taiwan

## Abstract

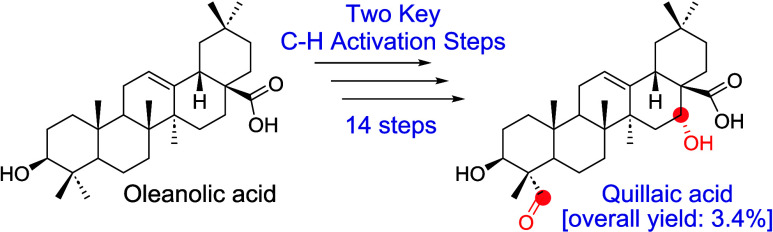

To meet the demand for quillaic acid, a multigram synthesis of quillaic acid was accomplished in 14 steps, starting from oleanolic acid, leading to an overall yield of 3.4%. Key features include C–H activation at C-16 and C-23. Through Pd-catalyzed C–H acetoxylation, the oxidation at C-23 was observed as the major product, as opposed to at C-24. A copper-mediated C–H hydroxylation using O_2_ successfully afforded the single isomer, 16β-ol triterpenoid, followed by configuration inversion to the desired 16α-ol compound. In summary, with steps optimized and conducted on a multigram scale, quillaic acid could be feasibly acquired through C–H activation with inexpensive copper catalysts, promoting a more sustainable approach.

## Introduction

**Figure 1:**
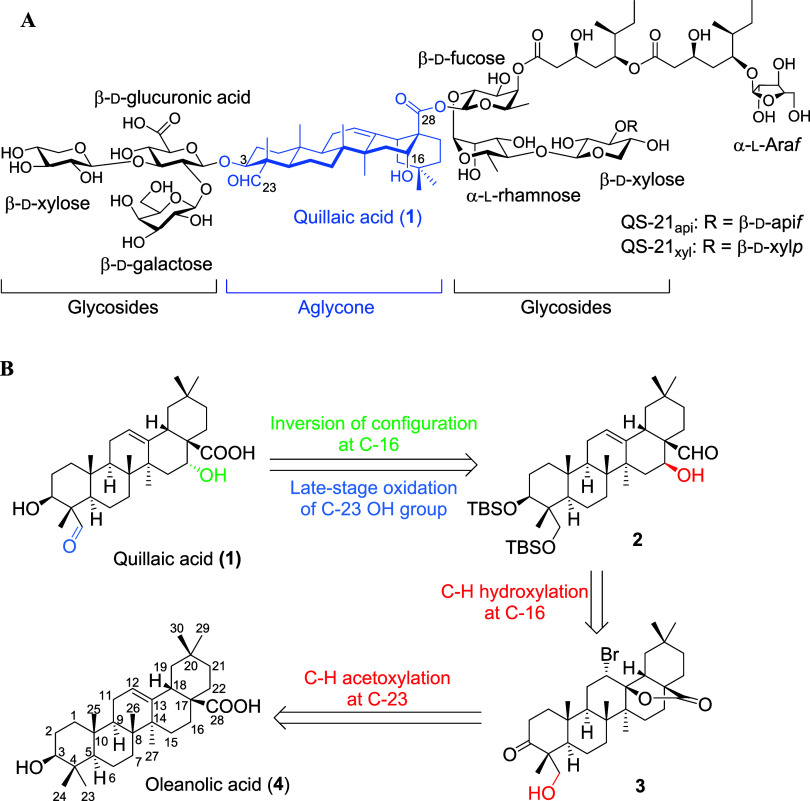
(A) Structure of triterpenoid, QS-21. (B) Our new approach to get quillaic acid (**1**).

Adjuvants, incorporated with vaccines, help spare dose and frequency of vaccines, accelerating effective immune responses.^1^ Saponin adjuvants, notably QS-21, have been extensively studied for their efficacy in veterinary and human vaccines. QS-21, renowned for its unique immunostimulatory properties, promotes a balanced Th1/Th2 immune response. This exceptional attribute has led to its inclusion in commercial vaccines, such as AS01_B_ in Shingrix for herpes zoster and AS01_E_ in Arexvy for respiratory syncytial virus vaccine, and several vaccines in clinical trials.^2^ Both AS01_B_ and AS01_E_ consist of QS-21, thus the rising demands for QS-21 in clinical vaccines have posed a challenge for the pharmaceutical industry, primarily stemming from the difficulty in achieving high-volume production of QS-21 due to its low-yield extraction from *Quillaja saponaria* extracts.^3^ Sources of its triterpenoid aglycone core, quillaic acid (**1**), hinder the stable supply of QS-21 through the semi- or total synthetic route (Figure 1A).^4^

Some methods have been devised to produce quillaic acid using easily accessible raw materials through biosynthetic or total synthetic approaches, aiming to reduce reliance on natural resources and to increase the economic viability of the compound.^5^ First, biosynthesis toward quillaic acid was proposed by introducing two novel cytochrome P450-dependent monooxygenases, CYP716A262 and CYP72A567, into the de novo biosynthesis pathway of oleanane-type triterpenoids.^5a^ Starting with β-amyrin using yeast strain BY-bAS with these CYP genes, the synthesis toward the oleanane-type aglycone could proceed successively, resulting in a strain production of 314.01 mg/L quillaic acid (**1**). Additionally, with the coexpression of β-amyrin synthase and CYP oxidases in*Nicotiana benthamiana*, the biosynthesis of quillaic acid was elucidated and offered the product.^5b^ Another biosynthesis was performed through combinatorial optimization of pairs of CYP monooxygenases and reductases to synthesize quillaic acid, using yeasts with spatial control of these enzymes on endoplasmic reticulum with the production of 2.23 g/L quillaic acid titer.^5c^ However, the advancement of biosynthetic techniques might face challenges in efficient cloning and precisely controlling the enzymes necessary for the process. On the other hand, the first chemical synthesis route to quillaic acid using protoescigenin as a starting material was developed in 2020. The key step of this process was iridium-catalyzed C–H activation at C-23, and the whole process was accomplished in 24 steps with a 4% overall yield.^5d^ Nevertheless, the synthetic procedure may be time-consuming and impractical for industrial applications. The pricing of echinocystic acid could only be scaled up to hundreds of milligrams, and hard-to-purify iridium in the synthetic routes might pose a possible industrial challenge.

To the best of our knowledge, except for these studies mentioned above, no further total chemical synthesis of quillaic acid has been revealed in the literature to date. Concerning the great progress in the research on C–H activation,^6^ C–H bond could be transformed efficiently under various conditions depending on reagents and chemical structures of substrates, especially for the regioselective C–H activation of triterpenoid.^7^ Herein, by adapting the idea of C–H activation and employing a combined strategy, we presented a total synthesis of quillaic acid (1), starting from oleanolic acid and proceeding in a more accessible way with each step performed on a multigram scale.

## Results and Discussion

Oleanolic acid (**4**) was selected and utilized as an ideal starting material in our synthetic strategy owing to its high abundance in the *Oleaceae* family, low cost, and low oxidation states (Figure 1B). From retrosynthetic analysis, quillaic acid (**1**) could be accessed through a configuration inversion and late-stage oxidation on the C-23 hydroxyl group in **2**. Importantly, the 16β-ol moiety of **2** would be constructed by selective C–H hydroxylation at C-16 of **3**. The acetoxy group at C-23 of **3** would be afforded by key selective C–H acetoxylation of oleanolic acid (**4**).

**Scheme 1:**
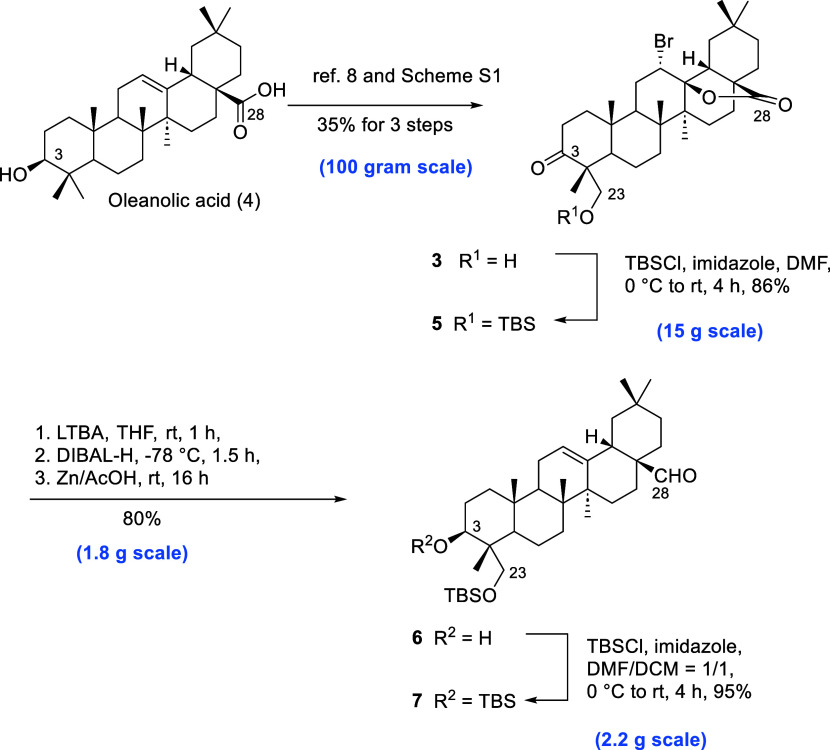
Process for the C–H Bond Activation to the C-23 Position of Oleanolic Acid (**4**)

Based on previous studies,^8,9^ compound **3** could be synthesized following known procedures from oleanolic acid (**4**), and subsequently, we scaled up the synthesis to multigrams (Scheme S1). Initial attempts at lactone opening and the reduction at C-3 of **3** failed under the direct treatment of lithium aluminum hydride (LAH),^8^ presumably attributed to the unprotected C-23 hydroxyl group. Hence, the C-23 hydroxyl group was protected by the *tert*-butyldimethylsilyl (TBS) group using *tert*-butyldimethylsilyl chloride (TBSCl) and imidazole in dimethylformamide (DMF) to afford **5** (86%; Scheme 1). To obtain the desired 3β-ol group by ketone reduction of **5**, stereoselectivity might be controlled through the small hydride reagents, such as lithium aluminum hydride (LAH) and lithium tri-*tert*-butoxyaluminum hydride (LTBA). Since LTBA possessed milder reactive properties than LAH, the C-28 ester bond was less susceptible to full reduction into the hydroxyl group. Consequently, compound **5** underwent ketone reduction using LTBA in tetrahydrofuran (THF) for 1 h to furnish the 3β-OH intermediate, followed by the successive treatment of diisobutylaluminum hydride (DIBAL-H) for the formation of the lactol intermediate at −78 °C. After the addition of Zn/AcOH for reduction at room temperature (rt),^8^ C-28 aldehyde **6** was afforded in 80% yield in this one-pot reaction (Scheme 1). To prevent the C-3 hydroxyl group of **6** from possible redox reactions in the following Cu-catalyzed C–H hydroxylation, the free hydroxyl group at C-3 of **6** was protected by the TBS group to give **7** in 95% yield.

**Scheme 2:**
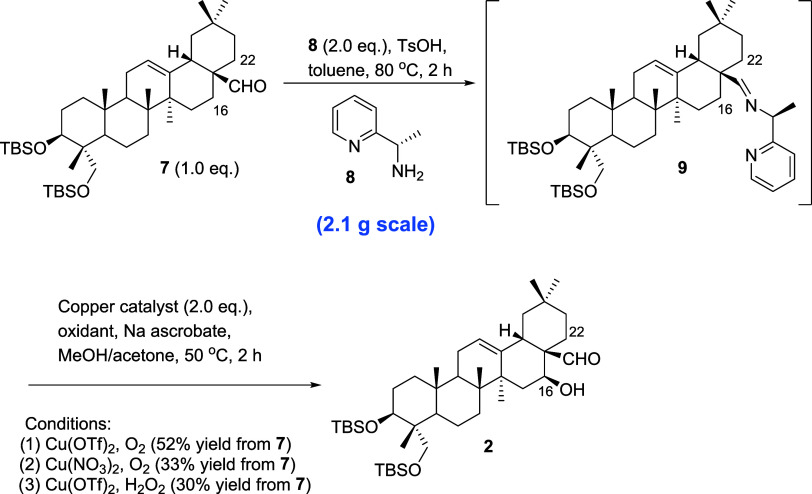
C–H Bond Activation to C-16 to 16β-ol Compound **2**

With the Cu-mediated C–H activation method reported previously,^7^ site-selective C–H hydroxylation could furnish 16β-ol or 22α-ol of pentacyclic triterpenoid when harnessing a transient directing group, (pyridine-2-yl) ethan-1-amine, suggesting that chiral directing groups oriented the position of the imine intermediate, particularly at either C-16 or C-22. As a result, (*S*)-1-pyridin-2-yl-ethylamine (**8**) was applied to compound **7** in the presence of *p*-toluenesulfonic acid (TsOH) in toluene at 80 °C, establishing imine–pyridine-conjugated intermediate **9** (Scheme 2). After the addition of Cu(OTf)_2_ and O_2_, the proposed complex^10^ of LCu^II^(OOH) was directed to its position toward 16β-H to build a hydroxyl group at C-16, forming compound **2** as the only single isomer in a 52% yield (Scheme 2). The usage of O_2_ could be represented as an eco-friendly reagent and a less hazardous approach toward C–H hydroxylation. As an essential C–H activation step, we also attempted to screen another copper catalyst and oxidant to achieve higher yields; however, the use of Cu(NO_3_)_2_ or H_2_O_2_ produced a lesser amount of product **2** in 33% or 30% yield, respectively.

**Scheme 3:**
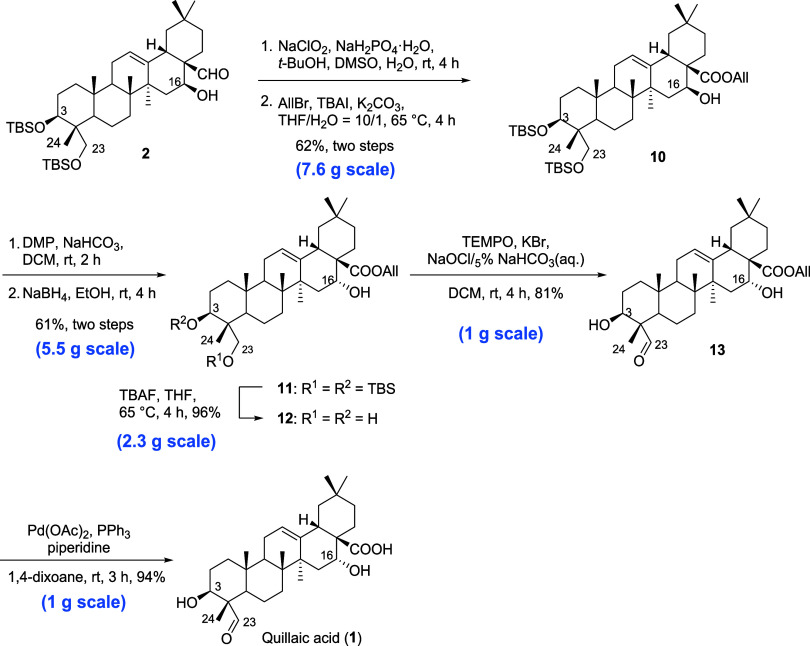
Synthesis of Quillaic Acid (**1**)

In order to obtain allyl-protected 16α-ol compound **11**, compound **2** was first subjected to Pinnick oxidation at C-28 through the oxidant NaClO_2_ under mildly acidic conditions of NaH_2_PO_4_ at rt for 2 h to oxidize C-28 aldehyde into a carboxylic acid (Scheme 3). Without further purification, the corresponding free carboxylic acid was subjected to allyl bromide in the presence of K_2_CO_3_ for 4 h to afford compound **10** in 62% yield (for two steps). As for the desired configuration of 16α–OH, which is crucial to the immune activity,^11^ we adopted the inversion strategy to extend the triterpene scaffold.^7,12^ Compound **10** was subjected to Dess–Martin oxidation using Dess–Martin periodinane (DMP) under the condition of NaHCO_3_ at rt for 2 h to give the C-16 ketone intermediate (Scheme 3). In the first attempt to selectively reduce the ketone intermediate, the reaction using LAH did not lead to the desired product, which might result from the steric hindrance of the structure. Therefore, a smaller nucleophile NaBH_4_ was utilized and subsequently achieved the inversion of configuration at C-16 into 16α-ol major product **11** in a 61% yield (for two steps). Reduction by using NaBH_4_ was first subjected to −78 °C,^7^ and we found that this reaction at room temperature also provided a satisfactory yield, which might be more practical in the scale-up process. The C3 and C23-*O*-silyl groups of **11** were removed under the treatment of tetrabutylammonium fluoride (TBAF), leading to compound **12** in an excellent yield of 96% (Scheme 3). As the late-stage oxidation, the selective oxidation of the primary alcohol to aldehyde was accomplished by 2,2,6,6-tetramethylpiperidinyloxy (TEMPO) oxidation, resulting in compound **13** with a yield of 81% (Scheme 3). After the deallylation under the conditions of Pd(OAc)_2_ and PPh_3_, quillaic acid (**1**) was afforded with an excellent yield of 94%. With the quillaic acid in hand, further reactions could be conducted to construct saponin analogues thereafter.

## Conclusions

In conclusion, starting from commercially available oleanolic acid, we have streamlined the chemical synthesis of quillaic acid from the previous 24 steps to 14 steps (Scheme S1) with an overall yield of 3.4% using two C–H activation steps to hydroxylate C-16 and C-23 and improved the feasibility of each step for potential industrial manufacturing in the future. By optimization of the appropriate conditions of C–H hydroxylation and other reactions, the reaction yield was enhanced, and side reactions, such as C-24 acetoxylation or C-22 hydroxylation, were minimized, rendering these reactions scalable. For C–H hydroxylation at C-16, regarding the essential catalysts utilized in C–H activation, 3d metal copper was adopted here for its inexpensive and less toxic properties compared to 4d metals. The utilization of molecular oxygen also gained wider application of this synthesis due to its nontoxic nature, inexpensiveness, and environmental sustainability as opposed to other oxidants. These results demonstrated that the synthetic route in this work successfully yielded quillaic acid on a gram scale, employing a sustainable strategy.

## Experimental Section

### General Information

All reagents and solvents were reagent grade and used without further purification unless otherwise noted. Those reagents that were stored in a refrigerator were opened and used after materials were warmed to rt. Molecular sieves were activated by heating at 200 °C and cooled to rt prior to use. Reaction progress was monitored by analytical thin-layer chromatography (TLC) on 0.25 mm Merck Millipore silica gel 60 F_254_ using *p*-anisaldehyde and cerium ammonium molybdate as staining agents. Flash column chromatography was performed using 230–400 mesh silica gel. Optical rotations were measured on a JASCO P-2000 polarimeter with [α]_D_^25^ values reported in deg dm^–1^ cm^3^ g^–1^, concentration (*c*) in g/100 mL. NMR spectra were acquired using Bruker-AV-400 (400 MHz) and Bruker-AV-600 (600 MHz) spectrometers. Structural assignments were made with additional information from the Correlation SpectroscopY (COSY), heteronuclear single quantum coherence (HSQC), and heteronuclear multiple bond correlation (HMBC) experiments. Chemical shifts (δ) are given in ppm relative to ^1^H: 7.26 ppm, ^13^C: 77.16 ppm for CDCl_3_; ^1^H: 3.31 ppm, ^13^C: 49.00 ppm for methanol-*d*_4_. Splitting patterns are reported as s (singlet), brs (broad singlet), d (doublet), brd (broad doublet), t (triplet), q (quartet), and m (multiplet). Coupling constants (*J*) are given in hertz (Hz). Reverse-phase high-performance liquid chromatography (HPLC) purification and analyses were carried out on a SHIMADZU HPLC system equipped with a system controller CBM-20A, a photodiode array detector SPD-M20A, a pump LC-20AT, and an autosampler SIL-20AHT. Exact mass measurements were performed on a VG platform electrospray ESI/MS or BioTOF II.

#### 23-*O*-*tert*-Butyldimethylsilyl-12α-bromo-13-hydroxy-3-oxo-oleanan-28-oic Acid-13-lactone (**5**)

To a stirred solution of white foam **3** (15.3 g, 27.9 mmol) in anhydrous DMF (60 mL) were added imidazole (5.7 g, 83.7 mmol) and TBSCl (10.6 g, 70.0 mmol) sequentially in an ice bath under a N_2_ atmosphere. After the solution was stirred at rt for 4 h, the mixture was diluted with EtOAc (750 mL) and washed with NaHCO_3(aq)_ (950 mL). The aqueous layer was extracted by EtOAc (750 mL) two times, and the organic layer was collected, dried over anhydrous MgSO_4_, filtered, concentrated under reduced pressure, and purified by column chromatography (silica gel, EtOAc/hexanes = 1:30) to give compound **5** (15.9 g, 86%) as white foam; R_*f*_ = 0.85 (EtOAc/hexanes = 1:2); [α]_D_^25^ +90.0 (*c* 1.31 in CHCl_3_); ^1^H NMR (600 MHz, CDCl_3_) δ 4.31 (dd, *J* = 3.7, 2.3 Hz, 1H), 3.59 (d, *J* = 9.3 Hz, 1H), 3.32 (d, *J* = 9.3 Hz, 1H), 2.53–2.47 (m, 1H), 2.46–2.37 (m, 2H), 2.36–2.33 (m, 1H), 2.20–2.14 (m, 2H), 2.05–1.91 (m, 5H), 1.91–1.86 (m, 2H), 1.67–1.56 (m, 6H), 1.52–1.47 (m, 1H), 1.46 (s, 3H), 1.39–1.28 (m, 5H), 1.27 (s, 3H), 1.26–1.23 (m, 1H), 0.99 (s, 3H), 0.91 (s, 3H), 0.90 (s, 3H), 0.89–0.88 (m, 9H), 0.04 (s, 3H), 0.01 (s, 3H) ppm; ^13^C{^1^H} NMR (151 MHz, CDCl_3_) δ 217.1, 178.7, 91.6, 68.6, 56.2, 52.4, 51.9, 45.8, 45.5, 44.2, 43.5, 42.1, 40.0, 37.3, 36.0, 35.6, 33.9, 33.6, 33.2, 31.9, 30.9, 29.1, 27.5, 25.8, 25.8, 25.8, 23.5, 21.3, 20.7, 19.0, 18.7, 18.2, 17.0, 16.5, −5.5, −5.8 ppm; high-resolution mass spectrometry (HRMS) (ESI-TOF) *m*/*z*: [M + H]^+^ calcd for C_36_H_60_BrO_4_Si, 663.3439; found, 663.3440.

#### 23-*O*-*tert*-Butyldimethylsilyl-3β-hydroxyoleanolic-28-aldehyde (**6**)

To a stirred solution of **5** (1.76 g, 2.66 mmol) in anhydrous THF (30 mL) was added lithium tri*-tert*-butoxyaluminum hydride (1.72 g, 6.76 mmol) at rt under a N_2_ atmosphere. After the reaction proceeded for 1 h, the mixture was cooled to −78 °C, and DIBAL-H (1.37 g, 9.63 mmol, 20 wt %) was added dropwise into the mixture. Upon completion of the reaction after 1.5 h, the reaction was quenched with MeOH (3.5 mL) dropwise at −78 °C. The resulting mixture was added to AcOH (30 mL) and Zn powder (2.7 g, 41.3 mmol) sequentially at rt for 1 min. After stirred at rt for 16 h, the mixture was diluted with EtOAc (60 mL), washed with water (50 mL) three times, dried over anhydrous MgSO_4_, filtered, concentrated under reduced pressure, and purified by column chromatography (silica gel, EtOAc/hexanes = 1:20) to give compound **6** (1.20 g, 80%) as white foam; R_*f*_ = 0.26 (EtOAc/hexanes = 1:4); [α]_D_^25^ +35.1 (*c* 1.32 in CHCl_3_); ^1^H NMR (600 MHz, CDCl_3_) δ 9.39 (s, 1H), 5.34 (t, *J* = 3.6 Hz, 1H), 3.66 (d, *J* = 9.4 Hz, 1H), 3.59 (dd, *J* = 10.7, 4.4 Hz, 1H), 3.35 (d, *J* = 9.3 Hz, 1H), 2.62 (dd, *J* = 13.7, 4.3 Hz, 1H), 1.97 (dt, *J* = 13.6, 4.1 Hz, 1H), 1.89–1.86 (m, 2H), 1.70–1.64 (m, 2H), 1.63–1.50 (m, 6H), 1.46–1.38 (m, 3H), 1.31–1.23 (m, 5H), 1.21–1.17 (m, 2H), 1.13 (s, 3H), 1.07–1.03 (m, 1H), 0.94 (s, 3H), 0.91–0.90 (m, 7H), 0.90 (s, 9H), 0.86 (s, 3H), 0.073 (s, 3H), 0.070 (s, 3H), 0.067 (s, 3H) ppm; ^13^C{^1^H} NMR (151 MHz, CDCl_3_) δ 207.5, 142.8, 123.3, 76.7, 73.2, 49.9, 49.1, 47.6, 45.6, 41.7, 41.6, 40.5, 39.5, 38.1, 36.8, 33.1, 33.1, 32.5, 30.6, 27.7, 26.7, 26.0, 25.8, 25.8, 25.8, 25.5, 23.4, 23.4, 22.1, 18.5, 18.1, 17.1, 15.5, 11.6, −5.7, −5.7 ppm; HRMS (ESI-TOF) *m*/*z*: [M + H]^+^ calcd for C_36_H_63_O_3_Si, 571.4541; found, 571.4544.

#### 3β,23-di-*O*-*tert*-Butyldimethylsilyloleanolic-28-aldehyde (**7**)

To a stirred solution of a white foam **6** (2.2 g, 3.86 mmol) in anhydrous DMF (12 mL) and dichloromethane (DCM, 12 mL) were added imidazole (0.67 g, 9.84 mmol) and TBSCl (1.16 g, 7.70 mmol) sequentially at 0 °C in an ice bath under a N_2_ atmosphere and then was warmed to rt. Upon completion of the reaction after 4 h, the mixture was diluted with EtOAc (120 mL) and washed with NaHCO_3(sat.)_ (150 mL). The aqueous layer was extracted by EtOAc (120 mL) two times. The organic layer was dried over anhydrous MgSO_4_, filtered, concentrated under reduced pressure, and purified by column chromatography (silica gel, EtOAc/hexanes = 1:200) to give **7** (2.5 g, 95%) as white foam; *R*_*f*_ = 0.52 (EtOAc/hexanes = 1:20); [α]_D_^25^ +24.9 (*c* 1.37 in CHCl_3_); ^1^H NMR (600 MHz, CDCl_3_) δ 9.40 (s, 1H), 5.34 (s, 1H), 3.70 (dd, *J* = 11.5, 4.8 Hz, 1H), 3.35 (d, *J* = 9.6 Hz, 1H), 3.15 (d, *J* = 9.6 Hz, 1H), 2.62 (dd, *J* = 13.7, 4.2 Hz, 1H), 1.96 (td, *J* = 13.7, 4.0 Hz, 1H), 1.89–1.85 (m, 2H), 1.72–1.66 (m, 2H), 1.65–1.60 (m, 2H), 1.56–1.52 (m, 3H), 1.51–1.42 (m, 3H), 1.33–1.27 (m, 4H), 1.26–1.17 (m, 5H), 1.11 (s, 3H), 1.10–1.06 (m, 1H), 0.92–0.91 (m, 8H), 0.90 (s, 9H), 0.86 (s, 9H), 0.73 (s, 3H), 0.57 (s, 3H), 0.03–0.02 (m, 12H) ppm; ^13^C{^1^H} NMR (151 MHz, CDCl_3_) δ 207.7, 142.8, 123.4, 71.6, 63.9, 49.1, 47.6, 45.9, 45.6, 43.2, 41.8, 40.6, 39.5, 38.1, 36.4, 33.2, 33.0, 32.2, 30.6, 27.7, 27.2, 26.7, 26.0, 26.0, 26.0, 25.9, 25.9, 25.9, 25.3, 23.4, 23.4, 22.1, 18.1, 18.0, 17.9, 17.1, 15.6, 12.7, −3.7, −4.9, −5.3, −5.8 ppm; HRMS (ESI-TOF) *m*/*z*: [M + H]^+^ calcd for C_42_H_77_O_3_Si_2_, 685.5406; found, 685.5406.

#### 3β,23-di-*O*-*tert*-Butyldimethylsilyl-16β-hydroxyoleanolic-28-aldehyde (**2**)

To a stirred solution of **7** (2.06 g, 3.00 mmol) in anhydrous toluene (30 mL) were added (*S*)-1-pyridin-2-yl-ethylamine **8** (0.73 g, 5.98 mmol) and TsOH (51.7 mg, 0.3 mmol) at rt under a N_2_ atmosphere. The reaction mixture was warmed to 80 °C in an oil bath, stirred for 2 h, and concentrated under reduced pressure. The resulting mixture was dissolved in MeOH (15 mL) and acetone (15 mL), and then Cu(OTf)_2_ (2.17 g, 6.00 mmol) and sodium ascorbate (1.19 g, 6.01 mmol) were added at rt. An O_2_ balloon bubbled through the mixture for 0.5 h. Then, the mixture was warmed to 50 °C in an oil bath and stirred for 2 h. The reaction was diluted with EtOAc (30 mL), washed with Na_4_EDTA_(aq)(sat.)_ (30 mL), and stirred for 1 h. The aqueous layer was extracted with EtOAc (30 mL) three times. The organic layer was washed with brine (30 mL), dried over anhydrous MgSO_4_, concentrated under reduced pressure, and purified by column chromatography (silica gel, EtOAc/hexane = 1:40) to afford compound **2** (1.1 g, 52%) as a white solid; *R*_*f*_ 0.40 (EtOAc/hexanes = 1:10); [α]_D_^25^ +26.3 (*c* 1.03 in CHCl_3_); ^1^H NMR (400 MHz, CDCl_3_) δ 9.46 (s, 1H), 5.39 (s, 1H), 4.16 (d, *J* = 11.8 Hz, 1H), 3.68 (d, *J* = 7.8 Hz, 1H), 3.35 (d, *J* = 9.6 Hz, 1H), 3.14 (d, *J* = 9.5 Hz, 1H), 2.70 (d, *J* = 10.3 Hz, 1H), 1.97–1.94 (m, 1H), 1.86–1.79 (m, 3H), 1.61–1.50 (m, 11H), 1.37–1.27 (m, 6H), 1.17 (s, 3H), 0.95 (s, 3H), 0.91 (s, 6H), 0.89 (s, 9H), 0.85 (s, 9H), 0.76 (s, 3H), 0.57 (s, 3H), 0.02 (s, 12H) ppm; ^13^C{^1^H} NMR (151 MHz, CDCl_3_) δ 210.0, 141.7, 124.2, 71.6, 65.7, 63.9, 52.5, 46.7, 45.9, 45.3, 43.9, 43.3, 43.2, 39.7, 38.2, 36.7, 36.3, 33.1, 32.4, 32.1, 30.4, 27.2, 26.4, 26.0, 26.0, 26.0, 25.9, 25.9, 25.9, 23.5, 23.5, 21.7, 18.1, 18.0, 17.8, 17.2, 15.6, 12.7, −3.7, −4.9, −5.3, −5.9 ppm; HRMS (ESI-TOF) *m*/*z*: [M + H]^+^ calcd for C_42_H_77_O_4_Si_2_, 701.5355; found, 701.5354.

#### 28-*O*-Allyl-3β,23-di-*O*-*tert*-butyldimethylsilyl-16β-hydroxyoleanolate (**10**)

To a stirred solution of compound **2** (7.55 g, 10.8 mmol) in dimethyl sulfoxide (DMSO, 22 mL) and *t*-BuOH (99 mL) was added a solution of NaClO_2_ (6.96 g, 77.0 mmol) and NaH_2_PO_4_·H_2_O (9.2 g, 76.7 mmol) in water (52 mL) at rt. Upon completion of the reaction after 4 h, the mixture was quenched with 10% NaOH_(aq)_, and the pH was adjusted to the basic solution. The reaction mixture was extracted with hexanes. The aqueous phase was then acidified with 1 N HCl_(aq)_ (to pH 1) and extracted with DCM (200 mL). The organic layer was washed with brine (200 mL), dried over anhydrous MgSO_4_, and concentrated under reduced pressure. The resulting crude mixture was dissolved in THF/H_2_O = 10:1 (330 mL), and allyl bromide (1.9 mL, 21.5 mmol), tetra-*n*-butylammonium iodide (199 mg, 0.54 mmol), and K_2_CO_3_ (2.98 g, 21.6 mmol) were added sequentially at rt. The reaction mixture was heated to 65 °C in an oil bath and stirred for 4 h. The reaction was concentrated under reduced pressure to remove THF, and the solution was diluted with EtOAc (300 mL). The aqueous layer was extracted with EtOAc (100 mL) two times. The organic layer was collected and washed with brine (200 mL), dried over anhydrous MgSO_4_, concentrated under reduced pressure, and purified by column chromatography (silica gel, EtOAc/hexane = 1:40) to afford **10** (5.02 g, 62% for two steps) as a white solid; *R*_*f*_ 0.60 (EtOAc/hexanes = 1:8); [α]_D_^25^ +12.6 (*c* 1.02 in CHCl_3_); ^1^H NMR (600 MHz, CDCl_3_) δ 5.88 (ddd, *J* = 22.4, 10.8, 5.7 Hz, 1H), 5.33 (d, *J* = 17.3 Hz, 1H), 5.30 (t, *J* = 3.5 Hz, 1H), 5.24 (d, *J* = 10.6 Hz, 1H), 4.54 (ddd, *J* = 22.4, 13.3, 5.6 Hz, 2H), 4.14 (dd, *J* = 11.9, 4.4 Hz, 1H), 3.70 (dd, *J* = 11.6, 4.8 Hz, 1H), 3.36 (d, *J* = 9.7 Hz, 1H), 3.15 (d, *J* = 9.7 Hz, 1H), 3.03 (dd, *J* = 14.0, 4.6 Hz, 1H), 2.26 (dt, *J* = 13.3, 3.2 Hz, 1H), 1.86 (dd, *J* = 8.8, 3.4 Hz, 2H), 1.72–1.58 (m, 3H), 1.57–1.41 (m, 8H), 1.37–1.22 (m, 6H), 1.17 (s, 3H), 1.17–1.13 (m, 1H), 0.96 (s, 3H), 0.91 (s, 6H), 0.90 (s, 9H), 0.86 (s, 9H), 0.73 (s, 3H), 0.57 (s, 3H), 0.02 (s, 12H) ppm; ^13^C{^1^H} NMR (151 MHz, CDCl_3_) δ 177.9, 142.3, 131.8, 123.1, 118.4, 71.6, 65.1, 64.8, 63.9, 50.6, 46.8, 46.0, 45.5, 44.0, 43.3, 43.2, 39.4, 38.1, 37.4, 36.4, 33.3, 33.0, 32.1, 30.5, 27.2, 26.7, 26.7, 26.0, 26.0, 26.0, 25.9, 25.9, 25.9, 23.9, 23.5, 18.1, 18.0, 17.9, 17.0, 15.6, 12.7, −3.7, −4.9, −5.3, −5.9 ppm; HRMS (ESI-TOF) *m*/*z*: [M + H]^+^ calcd for C_45_H_81_O_5_Si_2_, 757.5617; found, 757.5621.

#### 28-*O*-Allyl-3β,23-di-*O*-*tert*-butyldimethylsilyl-16α-hydroxyoleanolate (**11**)

To a stirred solution of **10** (5.5 g, 7.3 mmol) in anhydrous DCM (73 mL) were added Dess–Martin periodinane (12.4 g, 29.2 mmol) and NaHCO_3_ (1.8 g, 21.9 mmol) at rt under a N_2_ atmosphere. After stirring for 2 h, the reaction was quenched with Na_2_SO_3(aq)(sat.)_. The reaction mixture was diluted with EtOAc (100 mL) and washed with Na_2_SO_3(aq)(sat.)_ (100 mL). The aqueous layer was extracted with EtOAc (100 mL) two times. The organic phase was collected, washed with brine (100 mL), dried over anhydrous MgSO_4_, filtered, and concentrated under reduced pressure. The resulting mixture was dissolved in EtOH (73 mL), and NaBH_4_ (2.8 g, 73.0 mmol) was added at rt. Upon completion of the reaction after 4 h, the reaction was quenched with H_2_O (100 mL), and then EtOH was removed under reduced pressure. The reaction mixture was diluted with DCM (100 mL), and the aqueous layer was separated and extracted with DCM (100 mL) three times. The combined organic layer was washed with brine (100 mL), dried over anhydrous MgSO_4_, concentrated under reduced pressure, and purified by column chromatography (silica gel, EtOAc/hexane = 1:40) to give **11** (3.36 g, 61% for two steps) as white foam; *R*_*f*_ = 0.44 (EtOAc/hexanes = 1:4); [α]_D_^25^ +18.2 (*c* 1.00 in CHCl_3_); ^1^H NMR (600 MHz, CDCl_3_) δ 5.90–5.83 (m, 1H), 5.40 (t, *J* = 3.6 Hz, 1H), 5.30 (ddd, *J* = 17.0, 4.5 Hz, 1H), 5.21 (ddd, *J* = 10.6, 3.8 Hz, 1H), 4.54–4.46 (m, 3H), 3.70 (dd, *J* = 11.4, 4.8 Hz, 1H), 3.35 (d, *J* = 9.7 Hz, 1H), 3.15 (d, *J* = 9.6 Hz, 1H), 3.07 (dd, *J* = 14.4, 4.4 Hz, 1H), 2.18–2.13 (m, 1H), 1.90–1.87 (m, 3H), 1.85–1.81 (m, 1H), 1.79–1.72 (m, 2H), 1.62–1.61 (m, 1H), 1.59–1.57 (m, 3H), 1.53–1.51 (m, 2H), 1.37 (dd, *J* = 15.1, 3.8 Hz, 1H), 1.32 (s, 3H), 1.29–1.28 (m, 1H), 1.26–1.25 (m, 3H), 1.21–1.11 (m, 3H), 0.97 (s, 3H), 0.93 (s, 3H), 0.90 (s, 12H), 0.86 (s, 9H), 0.73 (s, 3H), 0.57 (s, 3H), 0.03–0.02 (m, 12H) ppm; ^13^C{^1^H} NMR (151 MHz, CDCl_3_) δ 176.5, 142.5, 132.2, 123.2, 118.0, 75.1, 71.6, 65.1, 63.9, 48.9, 46.8, 46.4, 46.0, 43.2, 41.5, 40.8, 39.6, 38.1, 36.5, 35.5, 35.5, 32.8, 32.4, 30.6, 30.4, 27.2, 26.9, 26.0, 26.0, 26.0, 25.9, 25.9, 25.9, 24.6, 23.4, 18.1, 18.0, 17.9, 17.2, 15.8, 12.6, −3.7, −4.9, −5.3, −5.8 ppm; HRMS (ESI-TOF) *m*/*z*: [M + H]^+^ calcd for C_45_H_81_O_5_Si_2_, 757.5617; found, 757.5621.

#### 28-*O*-Allyl-3β,16α,23-trihydroxyoleanolate (**12**)

To a stirred solution of **11** (2.3 g, 3.0 mmol) in anhydrous THF (60 mL) was added TBAF (30 mL, 30.0 mmol, 1 M solution in THF) at rt under a N_2_ atmosphere, and the resulting mixture was heated to 65 °C and stirred for 4 h. The reaction solution was concentrated under reduced pressure to remove THF, diluted with DCM (80 mL), and quenched with H_2_O (80 mL). The organic layer was extracted with brine (80 mL), dried over anhydrous MgSO_4_, and concentrated under reduced pressure. The residue was purified by flash column chromatography (silica gel, EtOAc/hexane = 1:4) to give **12** (1.54 g, 96%) as white foam; *R*_*f*_ = 0.39 (EtOAc/hexanes = 1:1); [α]_D_^25^ +23.8 (*c* 0.46 in CHCl_3_); ^1^H NMR (600 MHz, CDCl_3_) δ 5.90–5.84 (m, 1H), 5.39 (t, *J* = 3.5 Hz, 1H), 5.30 (ddd, *J* = 17.0, 2.8, 1.1 Hz, 1H), 5.21 (ddd, *J* = 10.6, 2.7, 1.4 Hz, 1H), 4.54–4.46 (m, 3H), 3.73 (d, *J* = 10.3 Hz, 1H), 3.64 (dd, *J* = 9.2, 7.1 Hz, 1H), 3.44 (d, *J* = 10.3 Hz, 1H), 3.07 (dd, *J* = 14.5, 4.4 Hz, 1H), 2.15 (t, *J* = 13.0 Hz, 1H), 1.91–1.88 (m, 3H), 1.84–1.81 (m, 2H), 1.80–1.76 (m, 6H), 1.66–1.59 (m, 4H), 1.50–1.46 (m, 1H), 1.49–1.44 (m, 1H), 1.43–1.38 (m, 1H), 1.35 (s, 3H), 1.27–1.25 (m, 3H), 1.14–1.11 (m, 1H), 0.97 (s, 3H), 0.96 (s, 3H), 0.91 (s, 3H), 0.89 (s, 3H), 0.73 (s, 3H) ppm; ^13^C{^1^H} NMR (151 MHz, CDCl_3_) δ 176.4, 142.7, 132.2, 122.8, 118.1, 74.9, 72.1, 65.2, 49.9, 48.8, 46.7, 46.3, 41.8, 41.2, 40.6, 39.5, 38.2, 36.9, 35.5, 32.8, 32.7, 30.6, 30.4, 29.7, 29.7, 27.0, 26.8, 24.7, 23.3, 18.4, 17.1, 15.8, 11.4 ppm; HRMS (ESI-TOF) *m*/*z*: [M + H]^+^ calcd for C_33_H_53_O_5_, 529.3888; found, 529.3887.

#### 28-*O*-Allyl Quillate (**13**)

To a stirred solution of **12** (1.0 g, 1.9 mmol) in DCM (20 mL) were added TEMPO (1.5 g, 9.5 mmol) and KBr (22 mg, 0.19 mmol) at rt. A solution of NaOCl (849 mg, 11.4 mmol) in 5% NaHCO_3(aq.)_ (35 mM) was added. The reaction mixture was stirred vigorously at rt for 4 h. The resulting solution was diluted with DCM (30 mL), washed with H_2_O (30 mL) and brine (30 mL), dried over anhydrous MgSO_4_, and concentrated under reduced pressure. The residue was purified by flash column chromatography (silica gel, EtOAc/hexane = 1:8) to give **13** (807 mg, 81%) as a white foam; *R_f_* = 0.24 (EtOAc/hexanes = 1:4); [α]_D_^25^ +36.7 (*c* 0.60 in CHCl_3_); ^1^H NMR (600 MHz, CDCl_3_) δ 9.35 (s, H-23, 1H), 5.87–5.78 (m, all internal alkenyl CH, 1H), 5.36 (s, H-12, 1H), 5.27 (d, *J* = 17.1 Hz, all terminal alkenyl CH_a_, 1H), 5.18 (d, *J* = 10.4 Hz, all terminal alkenyl CH_b_, 1H), 4.57–4.39 (m, H-16, allylic CH_2_, 3H), 3.75 (dd, *J* = 11.3, 4.1 Hz, H-3, 1H), 3.03 (dd, *J* = 14.2, 3.5 Hz, H-18, 1H), 2.14 (t, *J* = 13.6 Hz, H-19, 1H), 2.08–1.82 (m, 5H), 1.81–1.56 (m, 7H), 1.55–1.40 (m, 2H), 1.38–1.28 (m, 4H), 1.28–1.20 (m, 2H), 1.17 (d, *J* = 8.7 Hz, 1H), 1.13–0.98 (m, 5H), 0.94–0.93 (m, 7H), 0.88 (s, 3H), 0.70 (s, 3H) ppm; ^13^C{^1^H} NMR (151 MHz, CDCl_3_) δ 207.0 (C-23), 176.3 (C-28), 142.8, 132.1, 122.5, 118.1, 74.9, 71.8, 65.2, 55.2, 48.7, 48.2, 46.6, 46.4, 41.4, 40.6, 39.9, 38.1, 36.0, 35.5, 35.4, 32.8, 32.3, 30.7, 30.4, 27.0, 26.1, 24.6, 23.3, 20.7, 17.0, 15.7, 8.9 ppm; HRMS (ESI-TOF) *m*/*z*: [M + H]^+^ calcd for C_33_H_51_O_5_, 527.3731; found, 527.3733.

#### Quillaic Acid (**1**)

To a stirred solution of **13** (1.0 g, 1.9 mmol) in 1,4-dioxane (50 mL) were added Pd(OAc)_2_ (43 mg, 0.19 mmol), PPh_3_ (249 mg, 0.95 mmol), and piperidine (375 μL, 3.8 mmol) at rt. The reaction mixture was stirred at rt for 3 h. The resulting solution was concentrated under reduced pressure. The residue was purified by flash column chromatography (silica gel, EtOAc/hexane = 1:3) to give **1** (873 mg, 94%) as white foam; *R_f_* = 0.33 (EtOAc/hexanes = 1:1); [α]_D_^25^ +61.8 (*c* 1.03 in MeOH); ^1^H NMR (600 MHz, CD_3_OD) δ 9.30 (s, H-23, 1H), 5.31 (t, *J* = 3.5 Hz, H-12, 1H), 4.46 (s, H-16, 1H), 3.77 (t, *J* = 7.1 Hz, H-3, 1H), 3.01 (dd, *J* = 14.4, 4.3 Hz, H-18, 1H), 2.30 (t, *J* = 13.3 Hz, 1H), 1.98–1.89 (m, 4H), 1.84 (dd, *J* = 14.9, 3.8 Hz, 1H), 1.80–1.74 (m, 2H), 1.74–1.67 (m, 3H), 1.62–1.47 (m, 2H), 1.41 (s, 3H), 1.36–1.32 (m, 2H), 1.26 (dt, *J* = 12.6, 2.9 Hz, 1H), 1.18–1.11 (m, 2H), 1.06–1.02 (m, 1H), 1.01 (s, 3H), 1.00 (s, 3H), 0.97 (s, 3H), 0.92–0.89 (m, 1H), 0.89 (s, 3H), 0.80 (s, 3H) ppm; ^13^C{^1^H} NMR (151 MHz, CD_3_OD) δ 208.6 (C-23), 181.1 (C-28), 145.2, 123.1, 75.2, 72.8, 56.8, 49.5, 48.0, 47.7, 42.7, 42.1, 41.0, 39.4, 37.0, 36.6, 36.2, 33.6, 33.4, 32.8, 31.4, 27.3, 27.0, 24.9, 24.4, 21.7, 17.7, 16.2, 9.4 ppm; HRMS (ESI-TOF) *m*/*z*: [M – H]^−^ calcd for C_30_H_45_O_5_, 485.3272; found, 485.3264.

## Data Availability

The data underlying this study are available in the published article and its online Supporting Information.

## Supporting Information Available

Synthetic scheme; experimental procedures; and ^1^H, ^13^C{^1^H}, COSY, HSQC, and HMBC NMR spectra (PDF)
