# Supplementary material for: Synthesis of Quillaic Acid through Sustainable C–H Bond Activations
Source: J Org Chem. 2024 Apr 10;89(8):5491–7. doi: 10.1021/acs.joc.3c02958 (PMC11040720; doi:10.1021/acs.joc.3c02958)
Supplement: Supplementary file 1 — jo3c02958_si_001.pdf [file jo3c02958_si_001.pdf]

Supporting information

Synthesis of Quillaic Acid through Sustainable C-H Bond Activations

Yi-Chi Wang, Cheng-Ru Chen, Chien-Yi Chen, Pi-Hui Liang*

School of Pharmacy, College of Medicine, National Taiwan University, Taipei 100, Taiwan.

*Corresponding author.
Email: phliang@ntu.edu.tw

Table of contents

General Information S-2
Synthetic Scheme of Quillaic Acid S-3
Optimization of Reaction Conditions S-4
^{1}H, $^{13}C\{1H\}$, COSY, HSQC, HMBC NMR spectra S-5

General Information

All reagents and solvents were reagent grade and used without further purification unless otherwise noted. Those reagents which were stored in refrigerator, were opened and used after materials were warmed to rt. Molecular sieves were activated by heating at 200 °C and cooled down to rt prior to use. Reaction progress was monitored by analytical TLC on 0.25 mm Merck Milipore silica gel 60 F_{254} using *p*-anisaldehyde and cerium ammonium molybdate as staining agents. Flash column chromatography was performed using 230-400 mesh silica gel. Optical rotations were measured on a JASCO P-2000 polarimeter with $[\alpha]_D^{25}$ values reported in deg dm^{-1} cm^3 g^{-1}, concentration (*c*) in g/100 mL. NMR spectra were acquired using Bruker-AV-400 (400 MHz) and Bruker-AV-600 (600 MHz) spectrometers. Structural assignments were made with additional information from COSY, HSQC, and HMBC experiments. Chemical shifts (δ) are given in ppm relative to ^{1}H: 7.26 ppm, ^{13}C: 77.16 ppm for $CDCl_3$; ^{1}H: 5.32 ppm, ^{13}C: 53.84 ppm for methanol-d_4. Splitting patterns are reported as s (singlet), brs (broad singlet) d (doublet), brd (broad doublet), t (triplet), q (quartet) and m (multiplet). Coupling constants (*J*) are given in Hertz (Hz). Reverse phase HPLC purification and analyses were carried out on a HITACHI D-2000 Elite HPLC system equipped with autosampler L-2200, UV detector L-2420 and pump L-2130 or a SHIMADZU HPLC system equipped with system controller CBM-20A, photodiode array detector SPD-M20A, pump LC-20AT and autosampler SIL-20AHT. Exact mass measurements were performed on VG platform electrospray ESI/MS or BioTOF II.

Synthetic Scheme of Quillaic Acid

Scheme S1. Chemical synthesis of quillaic acid from oleanolic acid

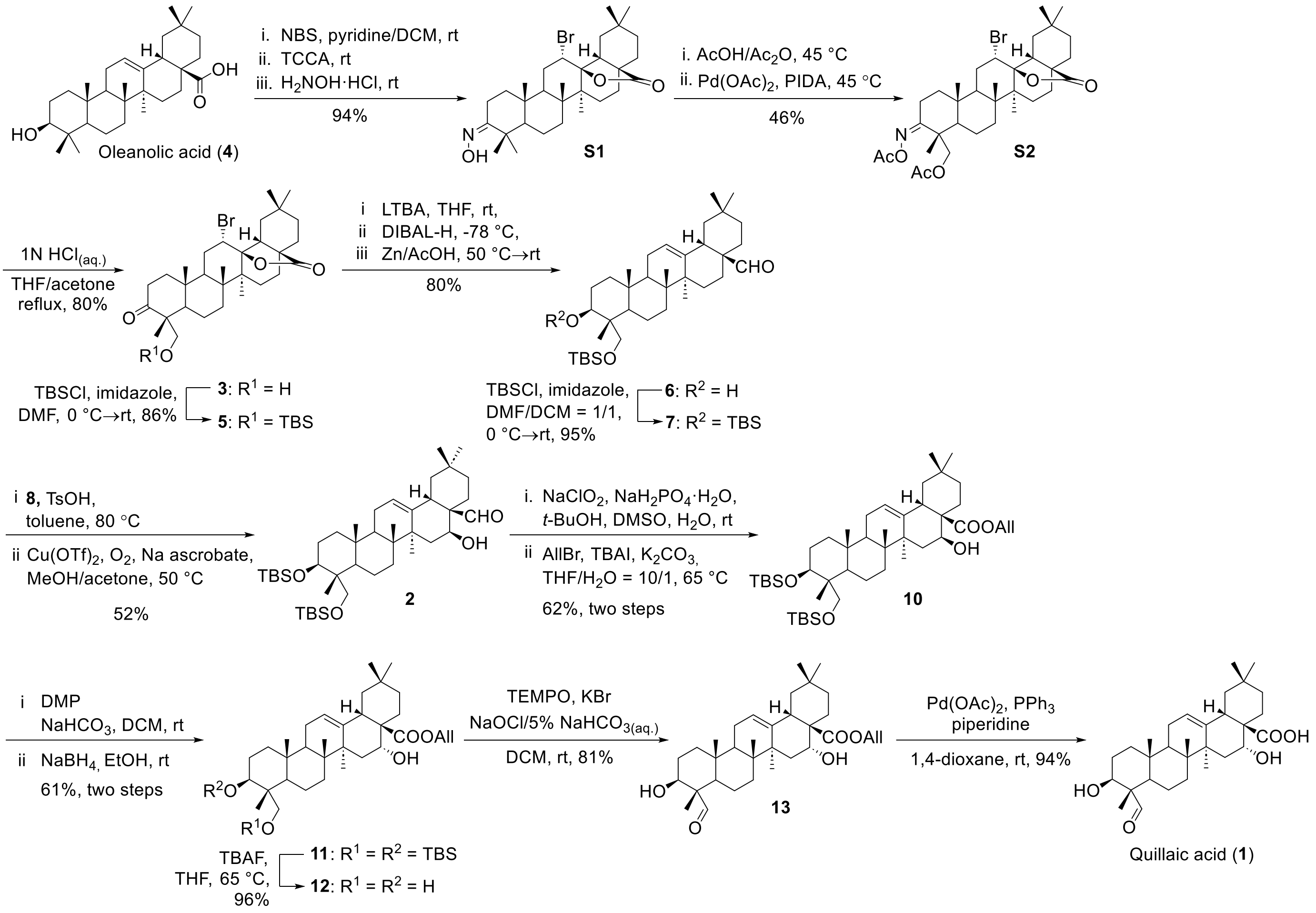

Optimization of Reaction Conditions

Table S1. Optimization of acetyl deprotection and deoximination of **S2**[a]

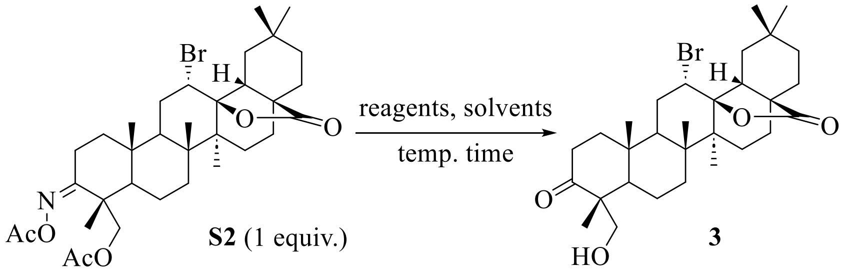

Entry	Reagents (equiv.)	Solvents	Temp.	Time	Yield[b]
1	$(PhCH_2Se)_2$ (5 mol%), H_2O_2 (45 mol%) $FeSO_4$ (1.25 mol%)	ACN	rt	8 h	9%
2	K_2CO_3 (0.5 equiv.)	MeOH	reflux	1.5 h	22%
	$CuSO_4{\cdot}5H_2O$ (5 equiv.)	ACN/H_2O	60 °C	20 h	
3	K_2CO_3 (0.5 equiv.)	MeOH	reflux	1.5 h	57%
	$CuSO_4{\cdot}5H_2O$ (5 equiv.)	ACN/H_2O	reflux	20 h	
4	K_2CO_3 (0.5 equiv.)	MeOH	reflux	1.5 h	71%
	$CuSO_4{\cdot}5H_2O$ (2.5 equiv.)	ACN/H_2O	reflux	12 h	
5	1 N $HCl_{(aq.)}$ (20 % wt/vol)	THF/Acetone	80 °C	8 h	61%
6	1 N $HCl_{(aq.)}$ (20 % wt/vol)	THF/Acetone	reflux	8 h	80%
7	2 N $HCl_{(aq.)}$ (20 % wt/vol)	THF/Acetone	reflux	8 h	76%
8	1 N $H_2SO_{4(aq.)}$ (20 % wt/vol)	THF/Acetone	80 °C	8 h	61%

[a]Reaction conditions were followed as below. Entry 1: To a stirred solution of **S2** (1 equiv.) in ACN (100 mM) was added $(PhCH_2Se)_2$ (5 mol%), H_2O_2 (0.45 equiv.) and $FeSO_4$ (1.25 mol%) at rt for 8 h; Entry 2–4: To a stirred solution of **S2** (1 equiv.) in MeOH (10 mM) was added K_2CO_3 (0.5 equiv.) heating to reflux for 1.5 h. After work-up procedure, the reaction mixture was dissolved in ACN/H_2O = 1/1 (50 mM) and added $CuSO_4{\cdot}5H_2O$ (2.5 equiv.) at 60 °C or heating to reflux, for 12 h or 20 h. Entry 5–8: **S2** (1 equiv.) was dissolved in THF/Acetone/Acid = 1/1/1 solution at 80 °C or heating to reflux for 8 h; [b]isolated yield of product **3**.

^{1}H, ^{13}C{1H}, COSY, HSQC, HMBC NMR spectra

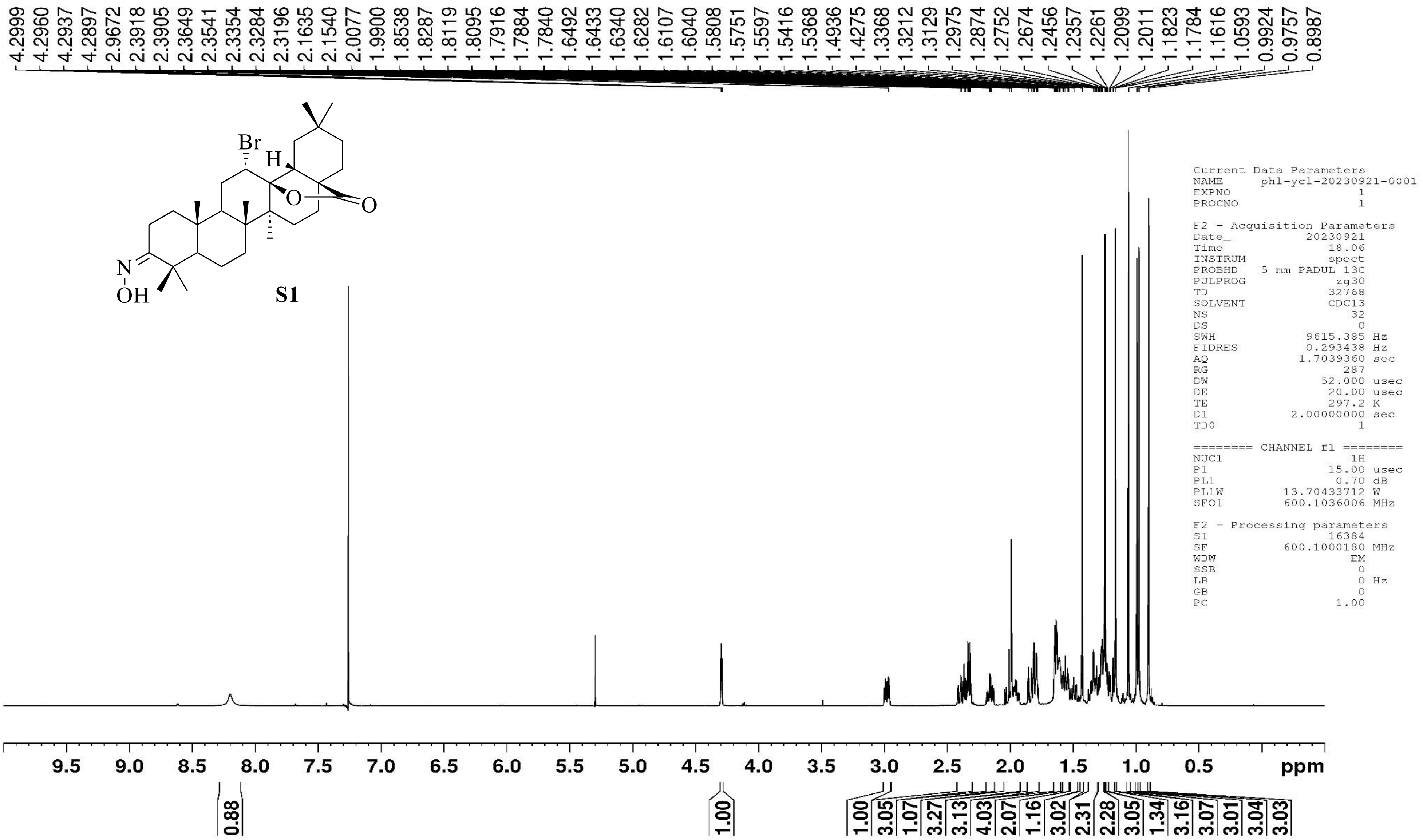

^{1}H NMR spectrum of **S1** ($CDCl_3$, 600 MHz).

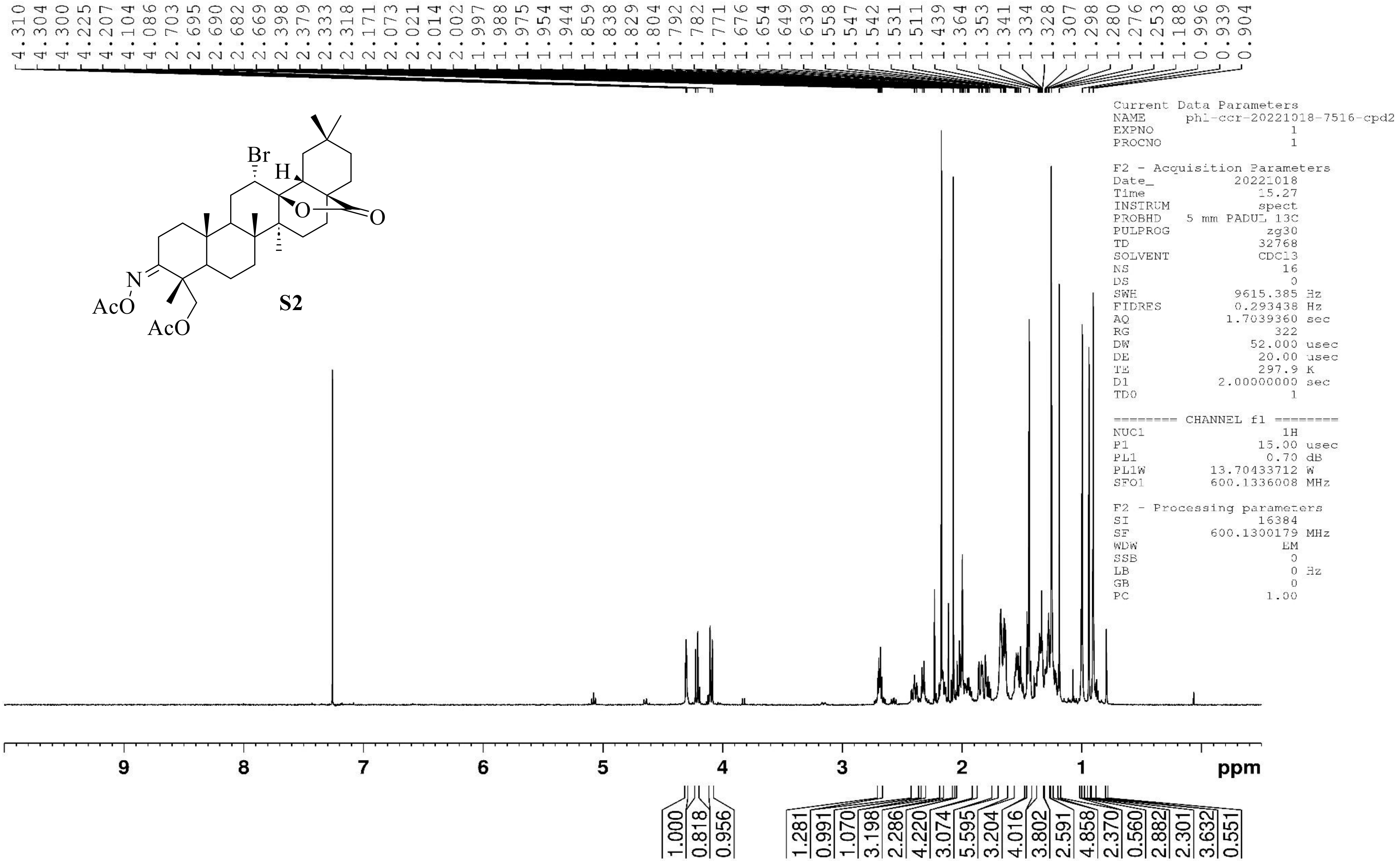

^{1}H NMR spectrum of **S2** ($CDCl_3$, 600 MHz).

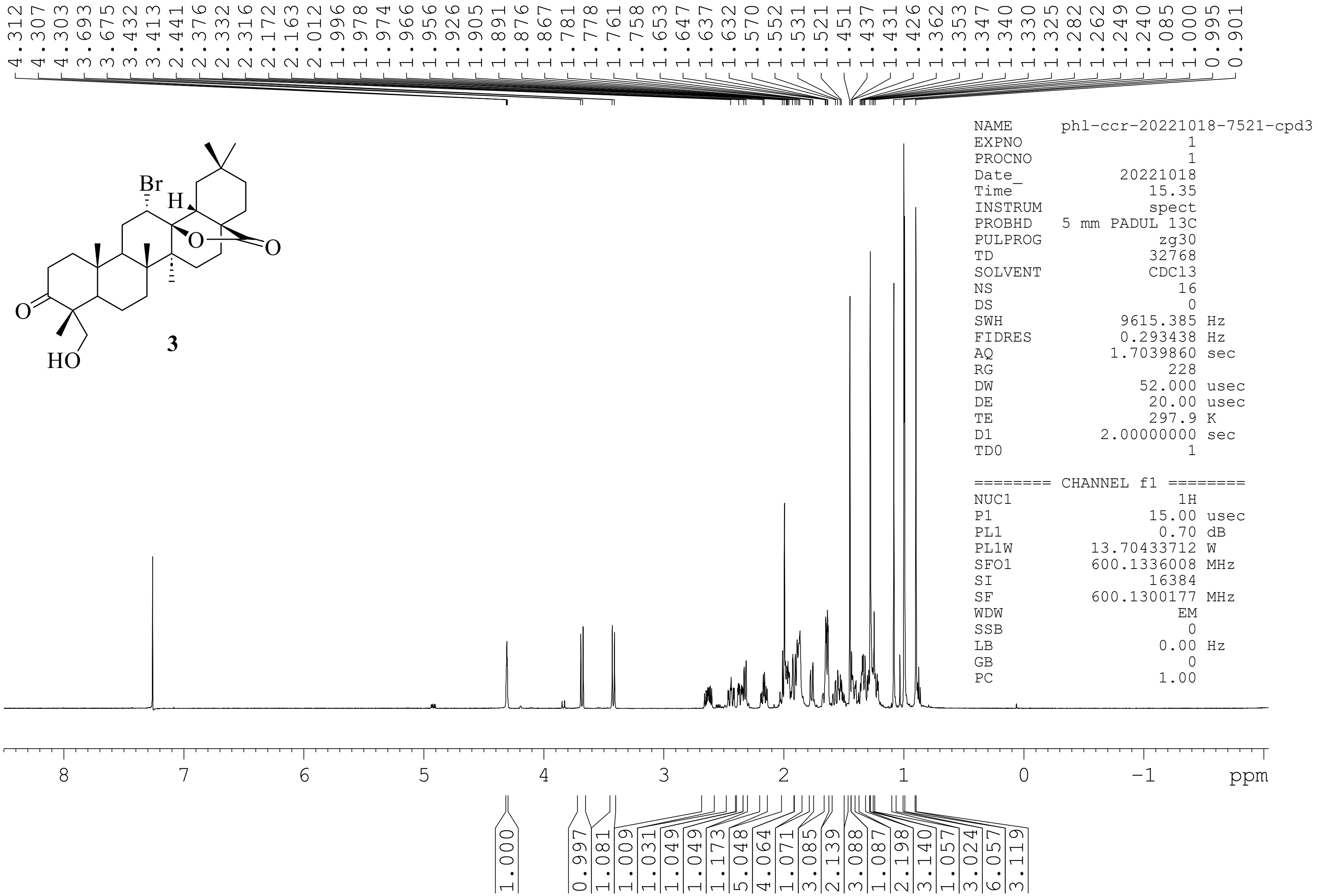

^{1}H NMR spectrum of **3** ($CDCl_3$, 600 MHz).

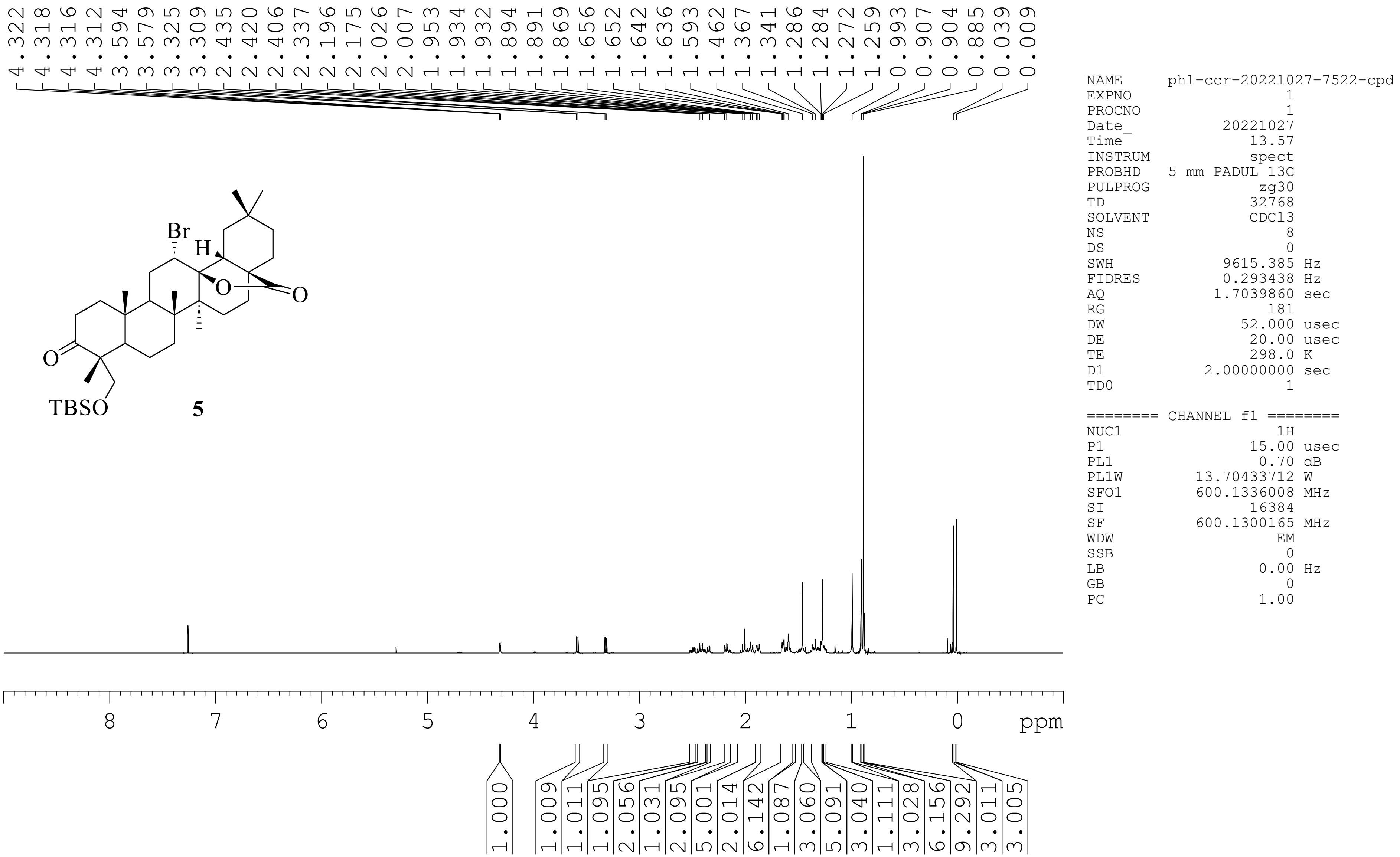

^{1}H NMR spectrum of **5** ($CDCl_3$, 600 MHz).

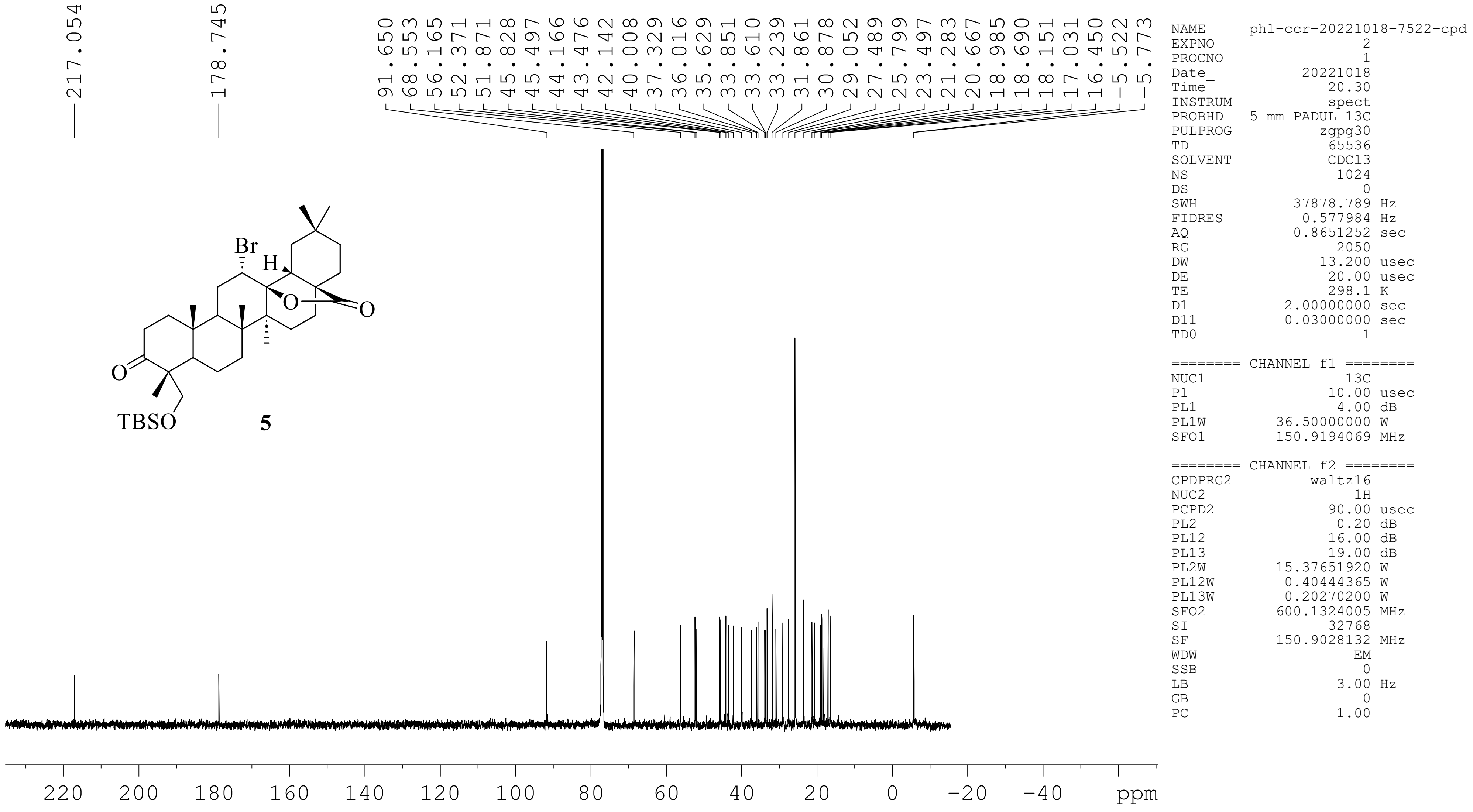

$^{13}C\{^{1}H\}$ NMR spectrum of **5** ($CDCl_3$, 151 MHz).

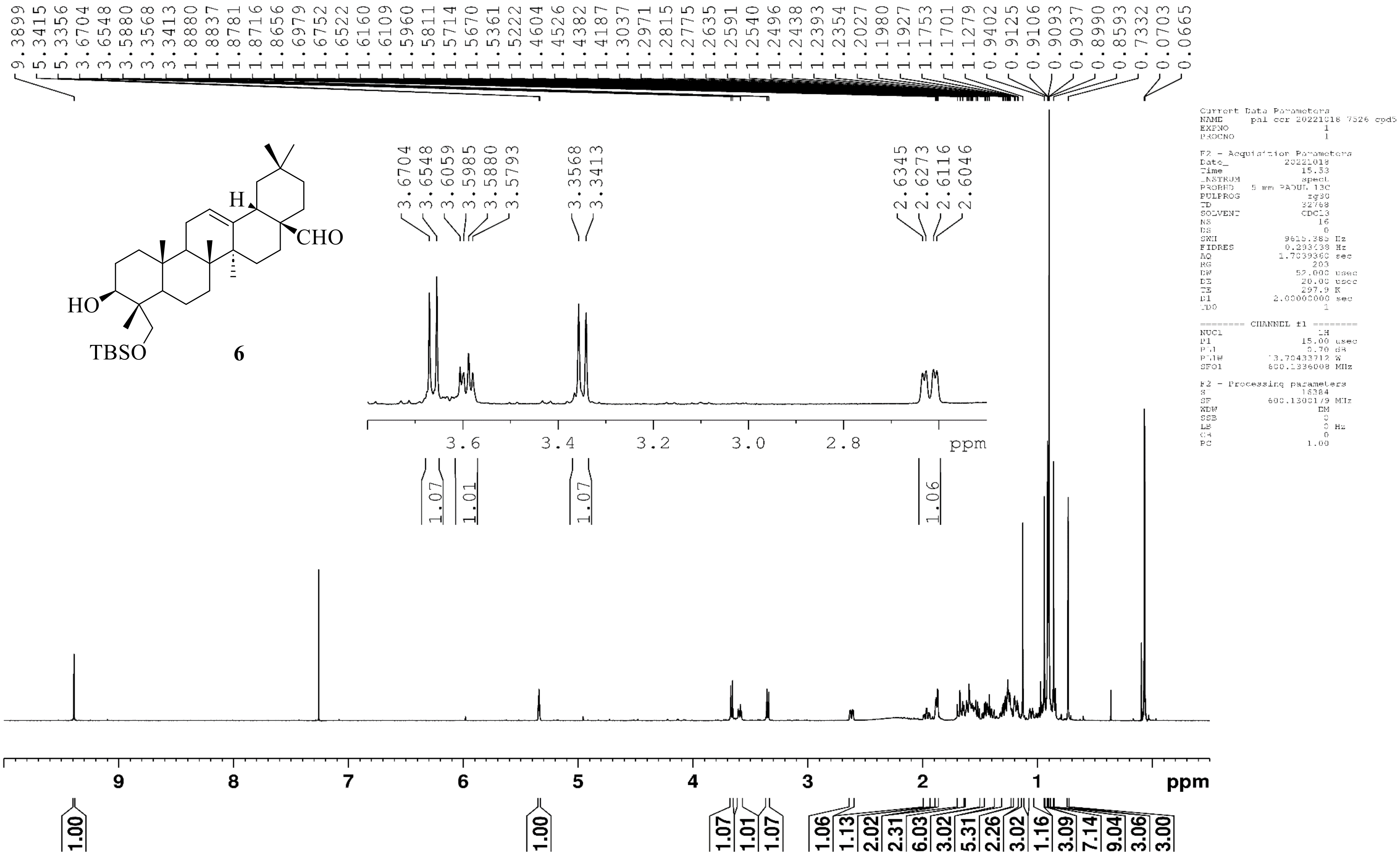

^{1}H NMR spectrum of **6** ($CDCl_3$, 600 MHz)

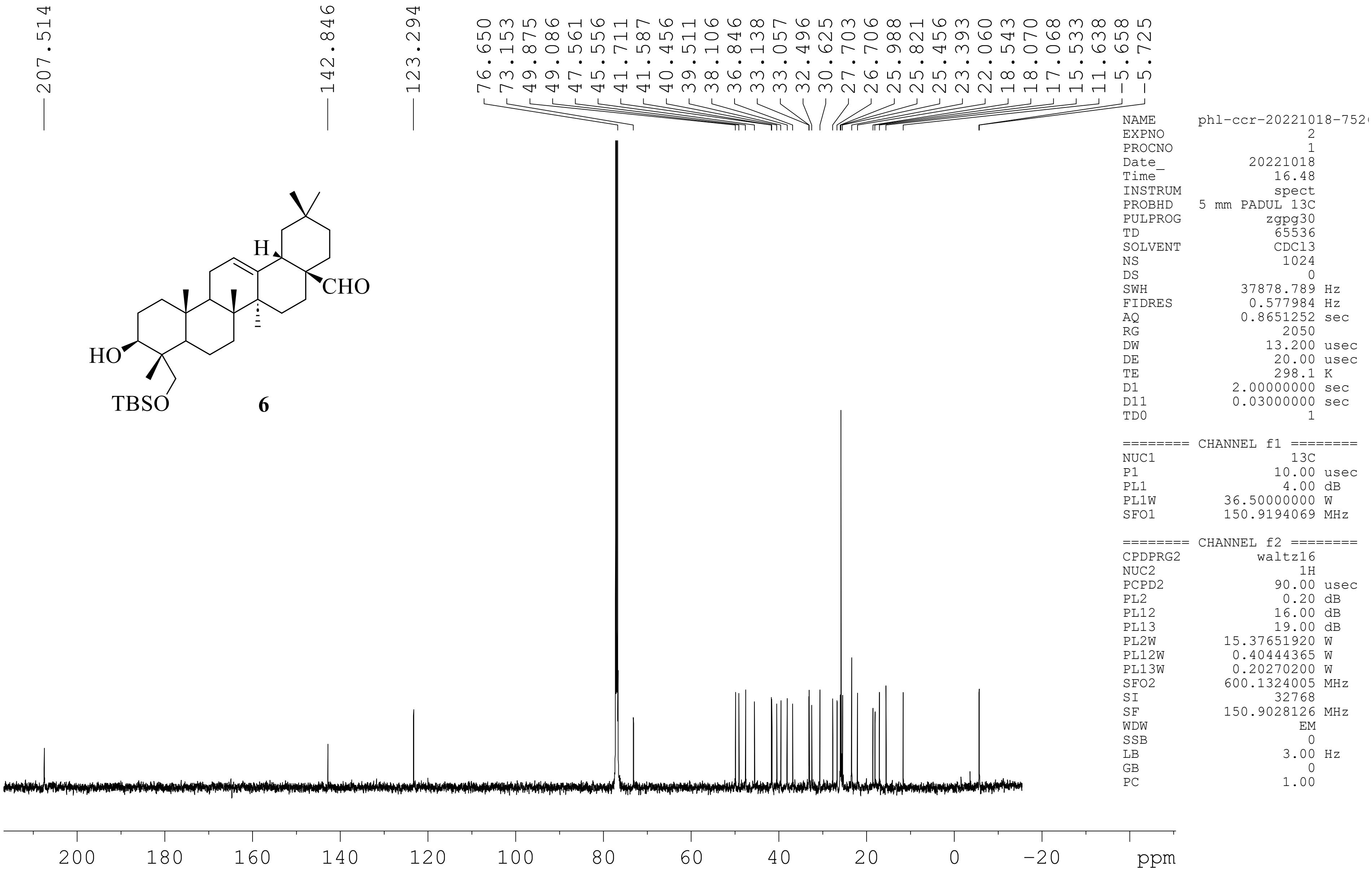

$^{13}C\{^1H\}$ NMR spectrum of **6** ($CDCl_3$, 151 MHz).

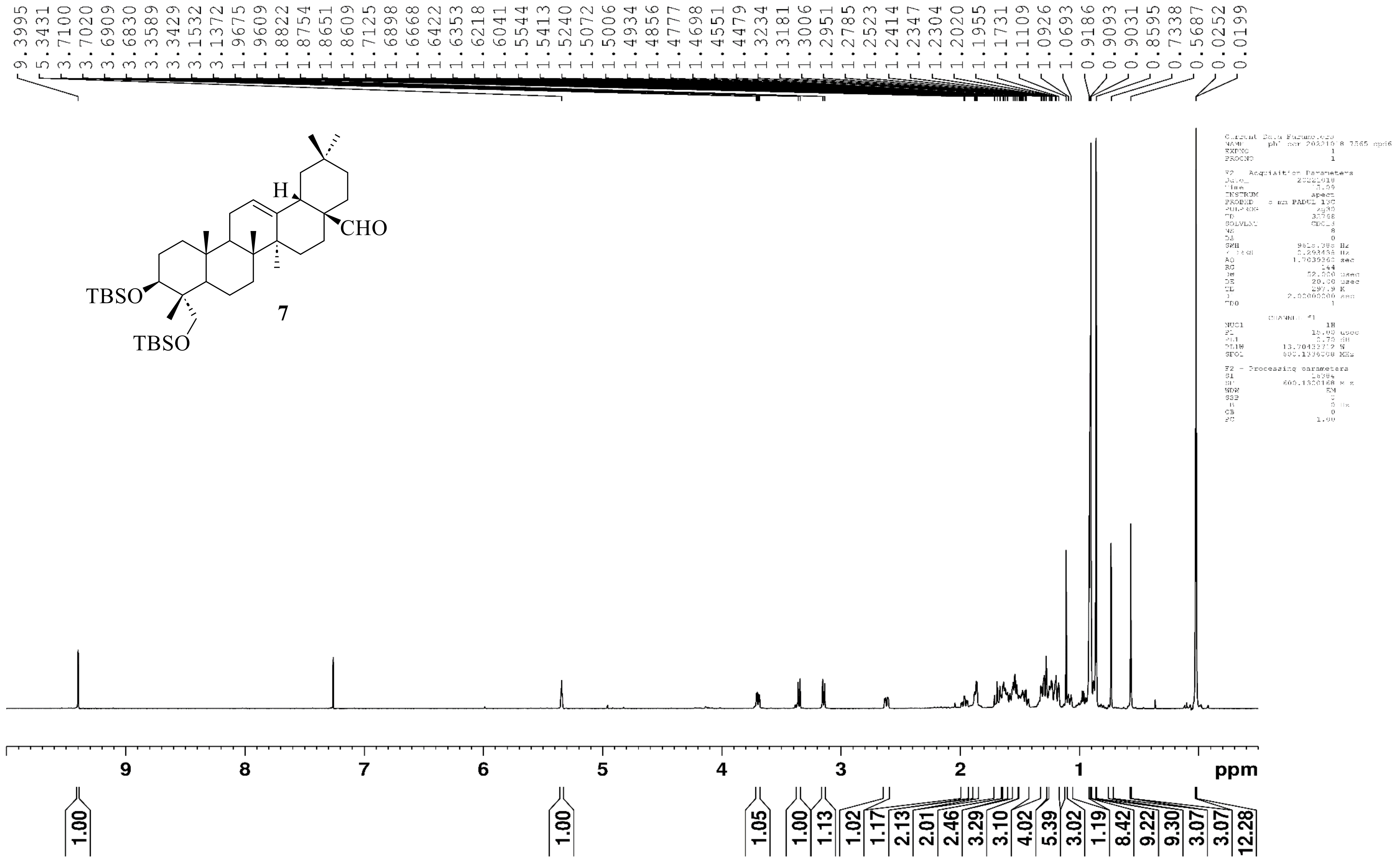

^{1}H NMR spectrum of **7** ($CDCl_3$, 600 MHz)

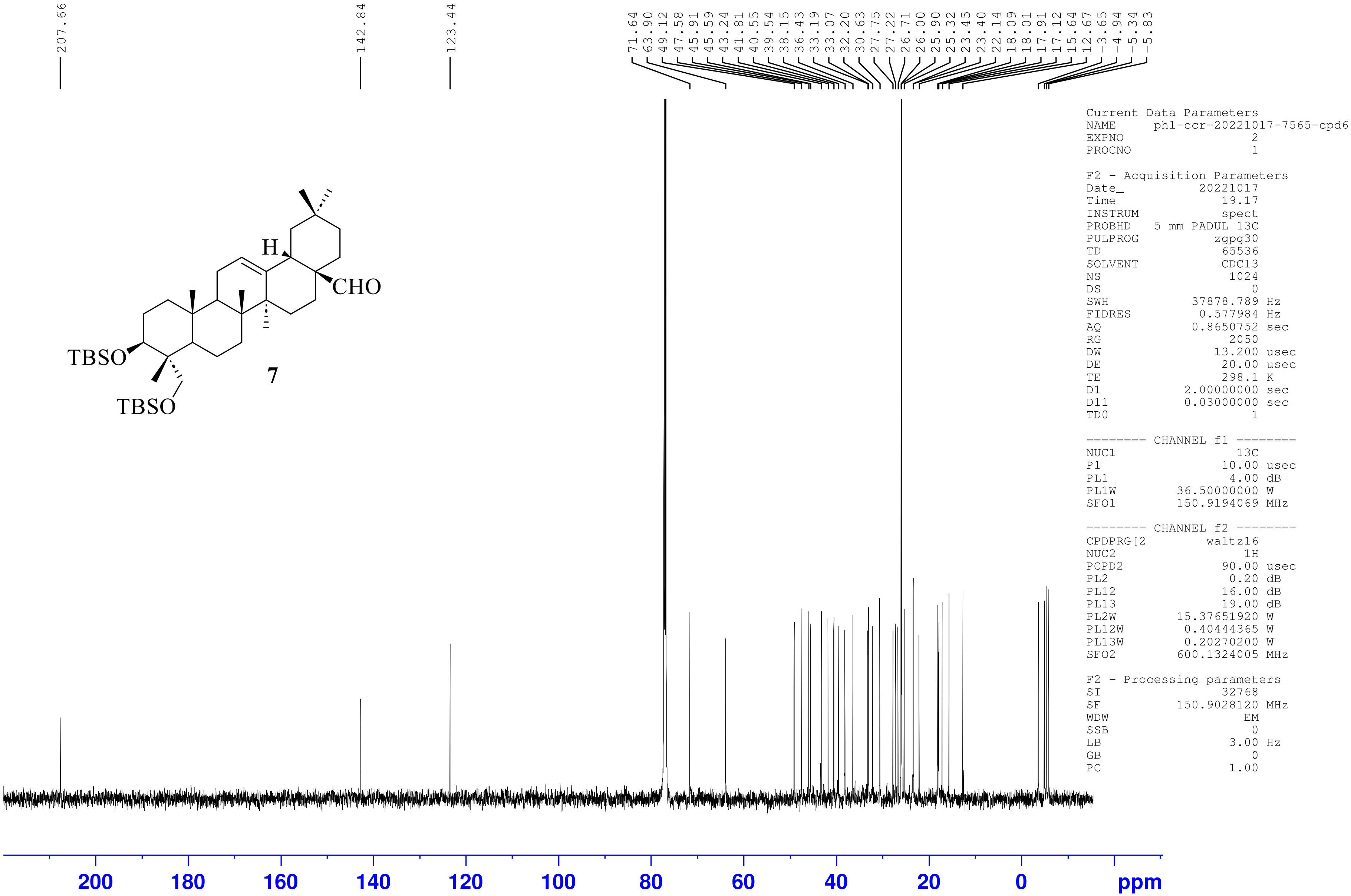

$^{13}C\{^{1}H\}$ NMR spectrum of **7** ($CDCl_3$, 151 MHz).

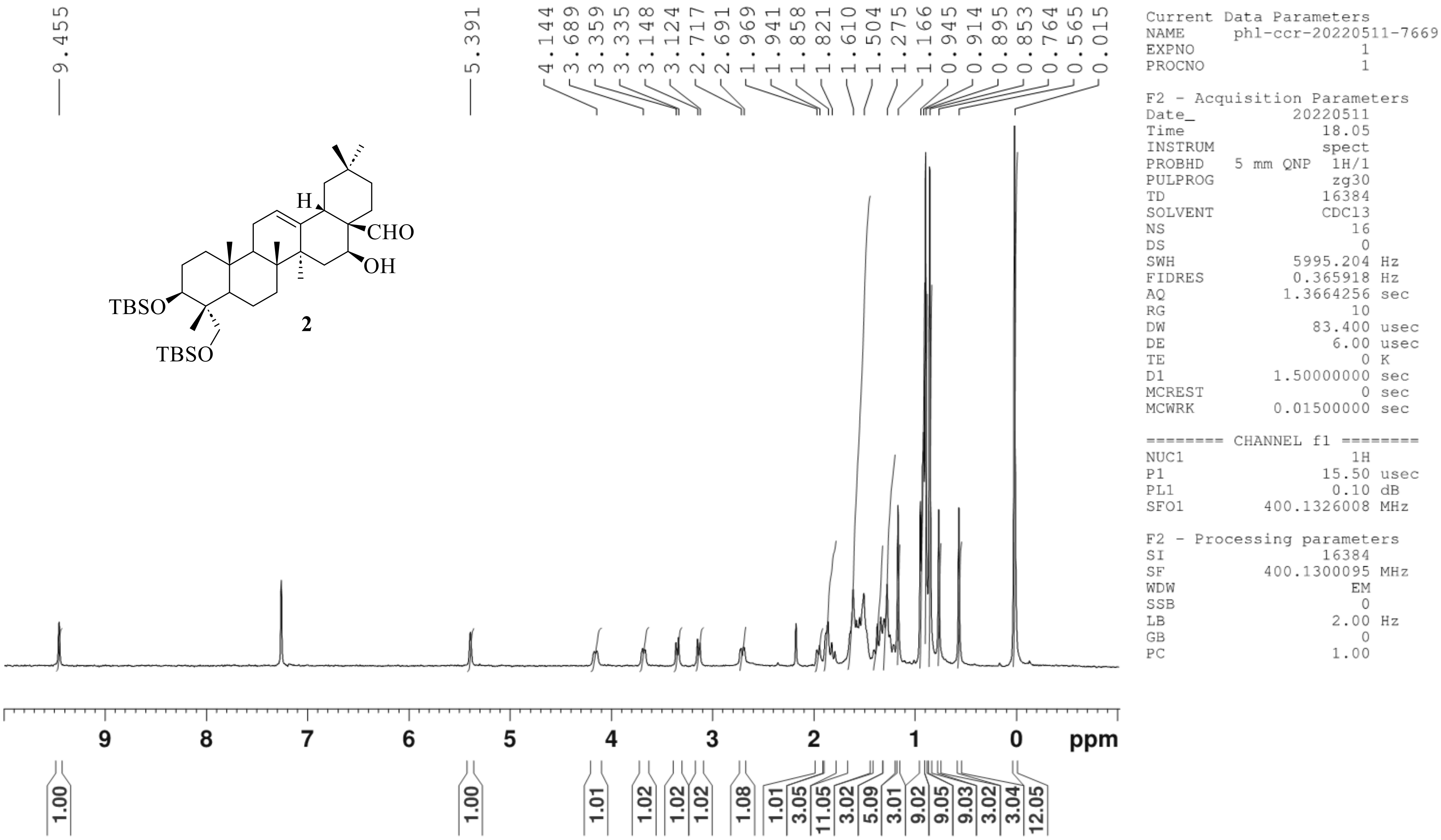

^{1}H NMR spectrum of **2** ($CDCl_3$, 400 MHz).

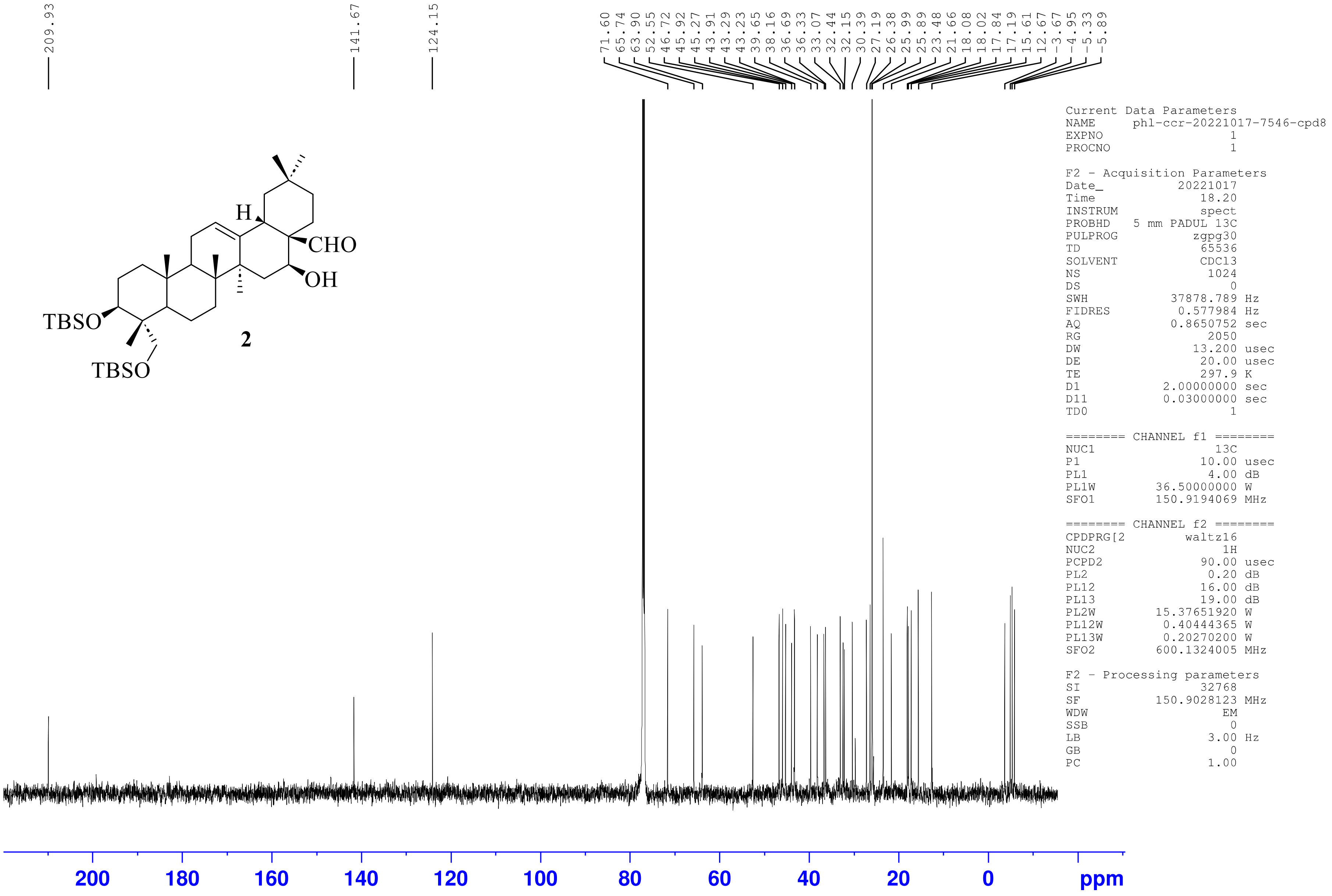

$^{13}C\{^{1}H\}$ NMR spectrum of **2** ($CDCl_3$, 151 MHz).

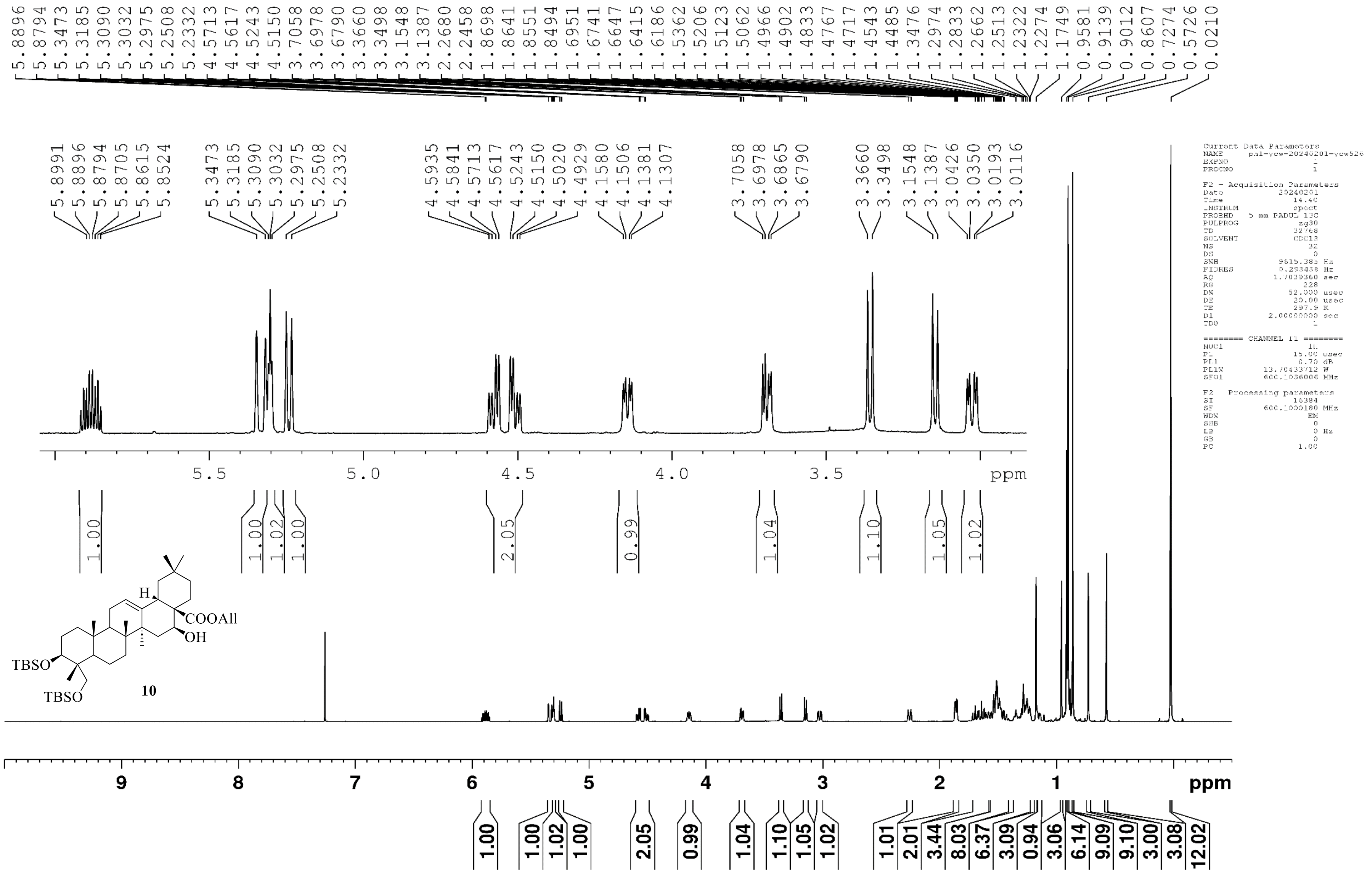

^{1}H NMR spectrum of **10** ($CDCl_3$, 600 MHz)

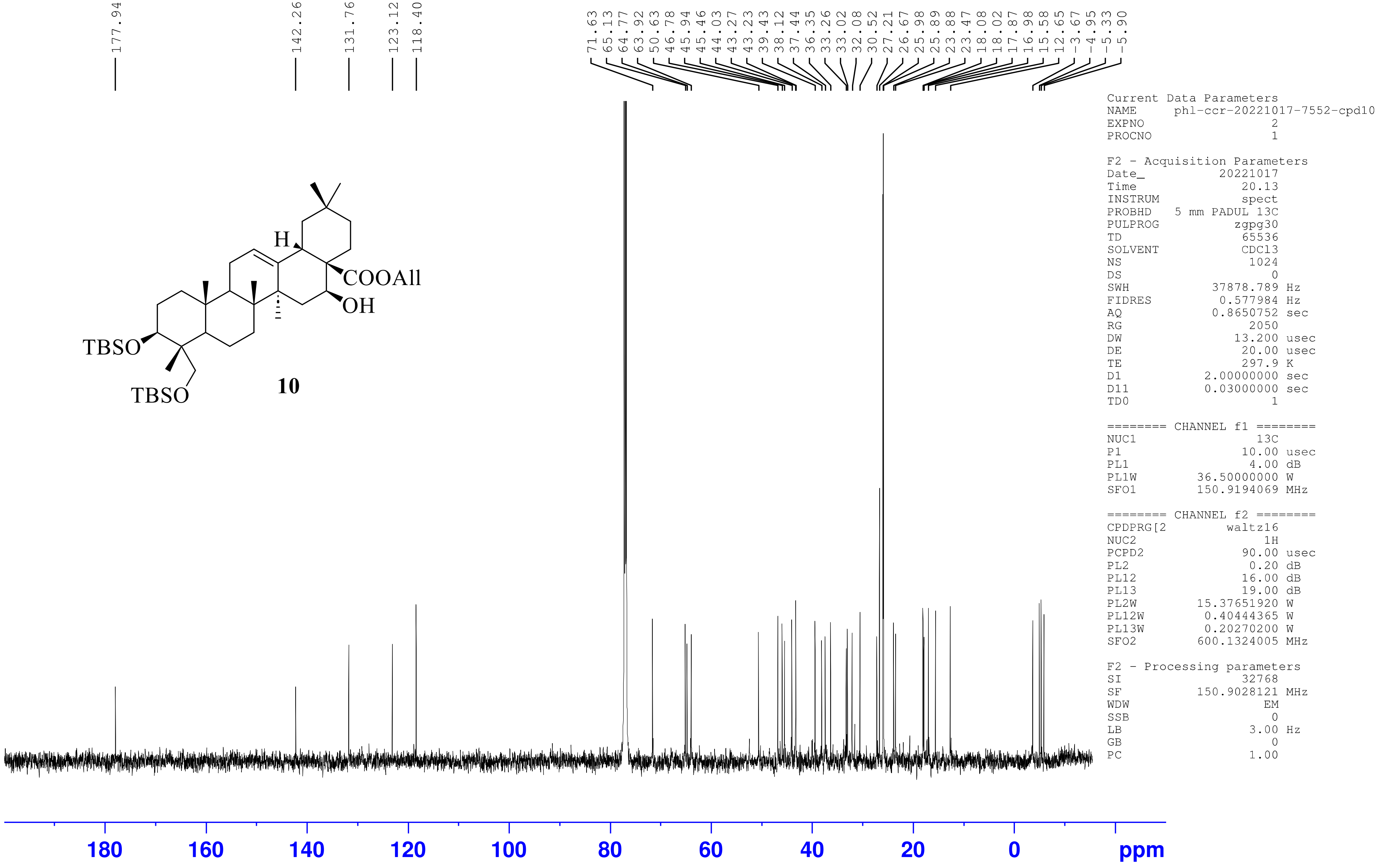

$^{13}C\{^{1}H\}$ NMR spectrum of **10** ($CDCl_3$, 151 MHz).

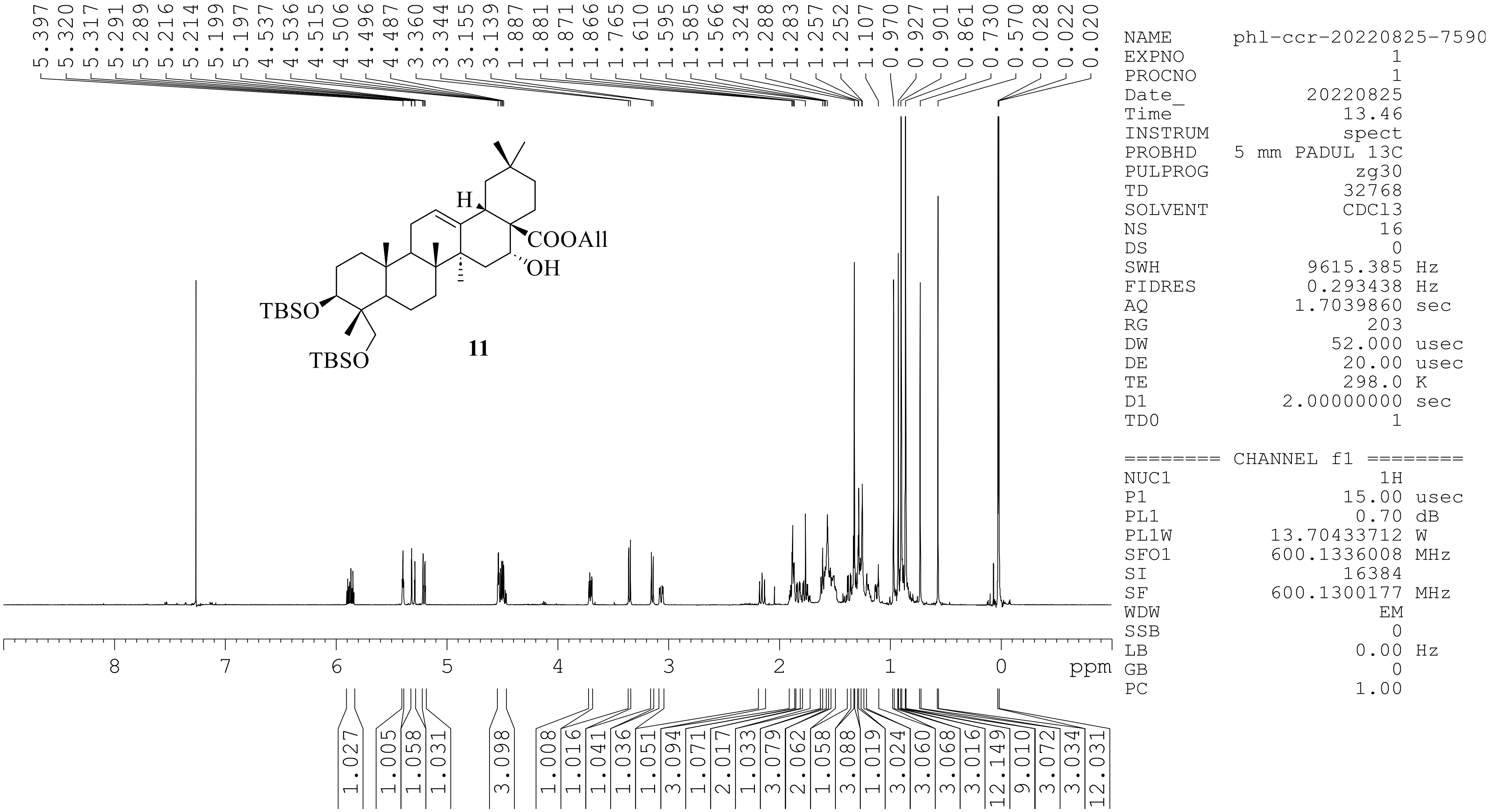

^{1}H NMR spectrum of **11** ($CDCl_3$, 600 MHz).

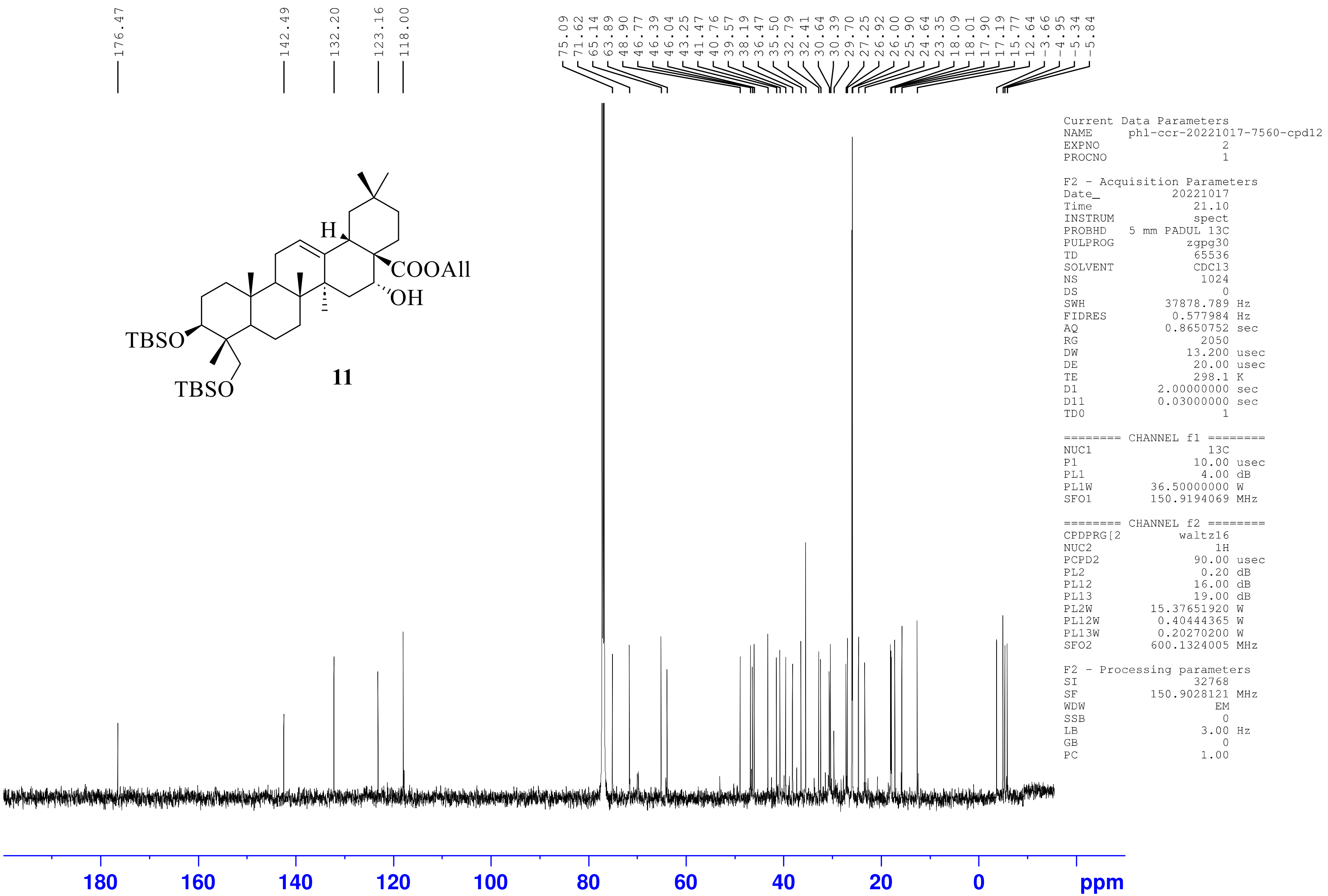

$^{13}C\{^{1}H\}$ NMR spectrum of **11** ($CDCl_3$, 151 MHz)

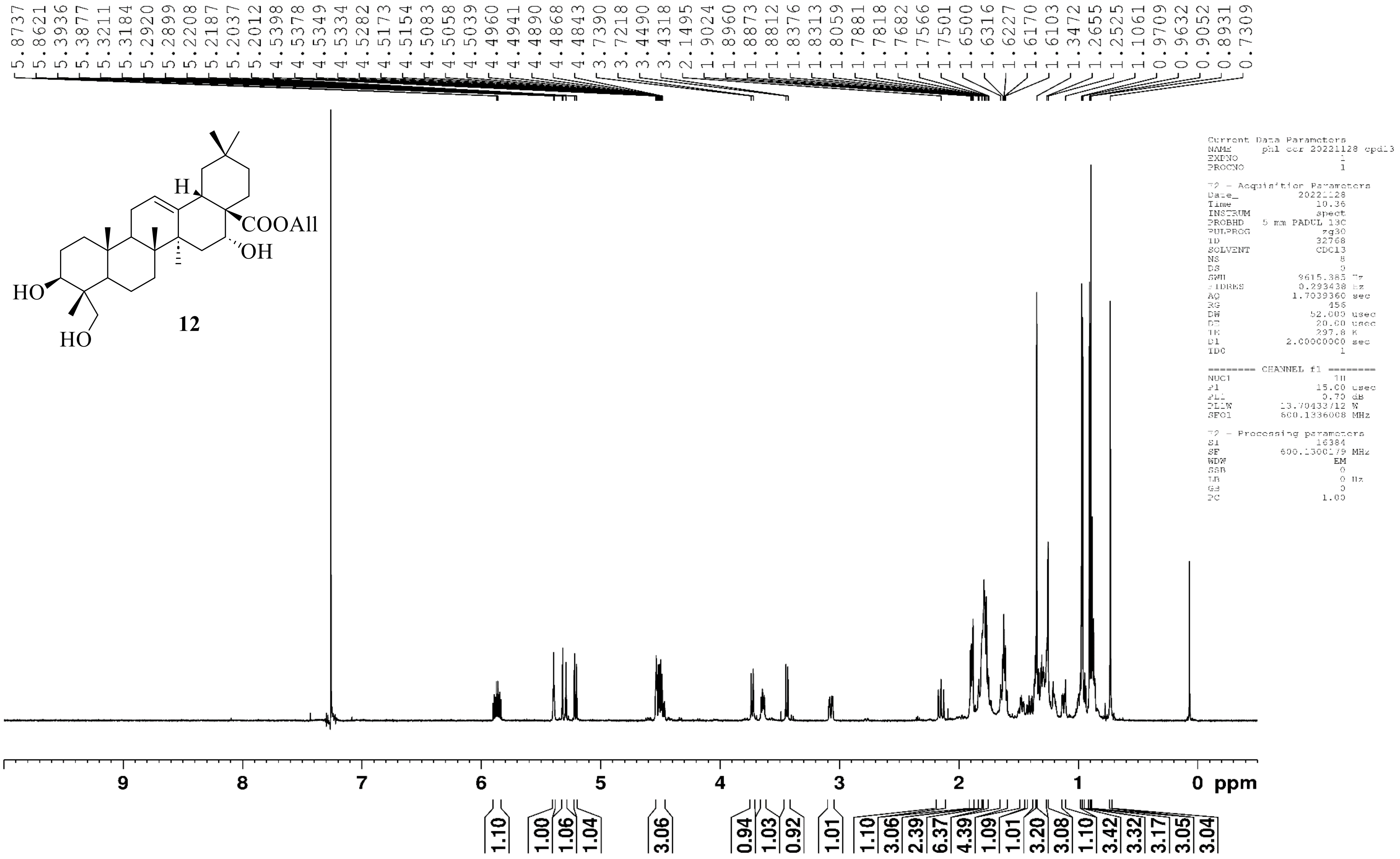

^{1}H NMR spectrum of **12** ($CDCl_3$, 600 MHz)

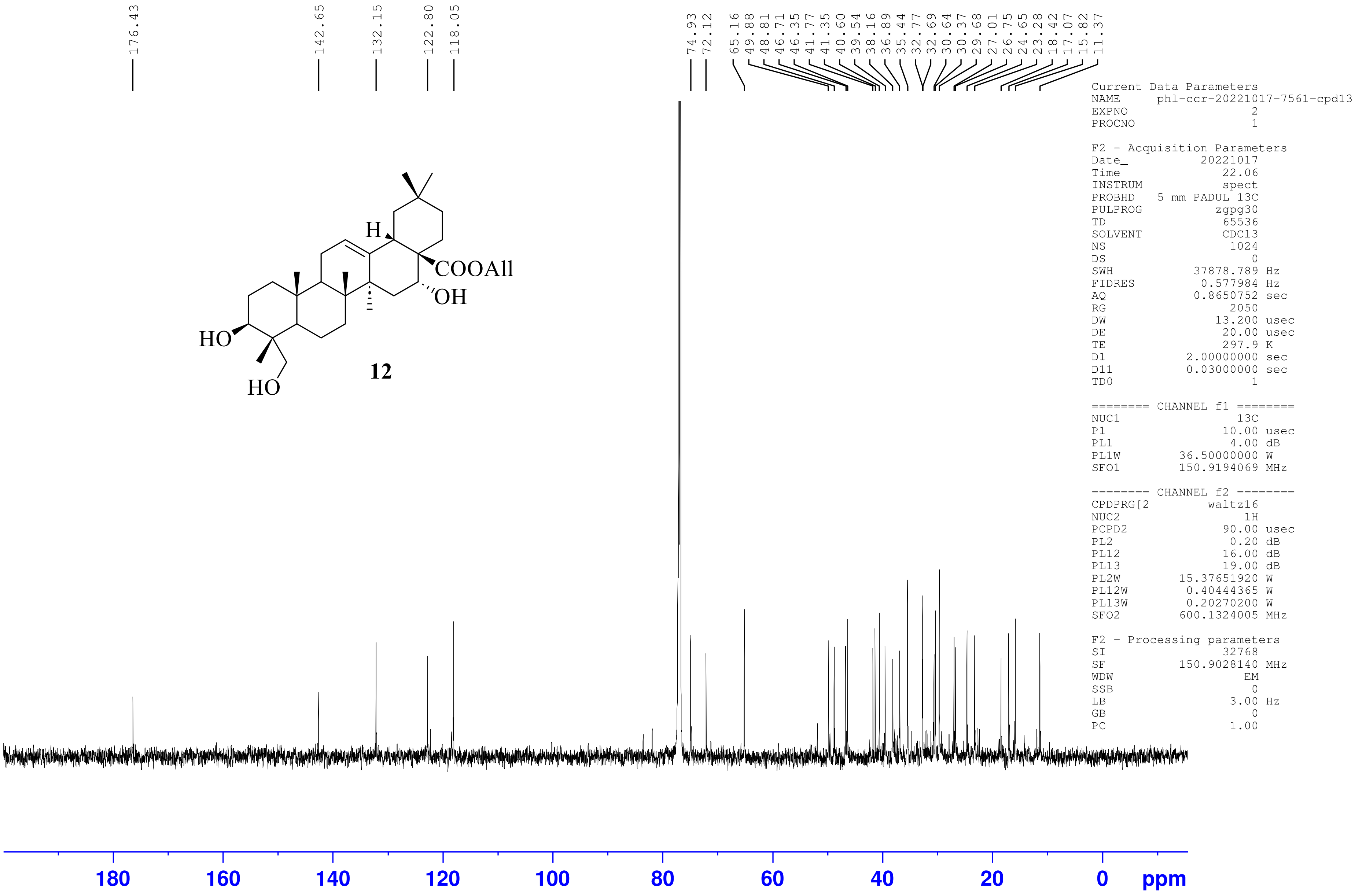

$^{13}C\{^{1}H\}$ NMR spectrum of **12** ($CDCl_3$, 151 MHz)

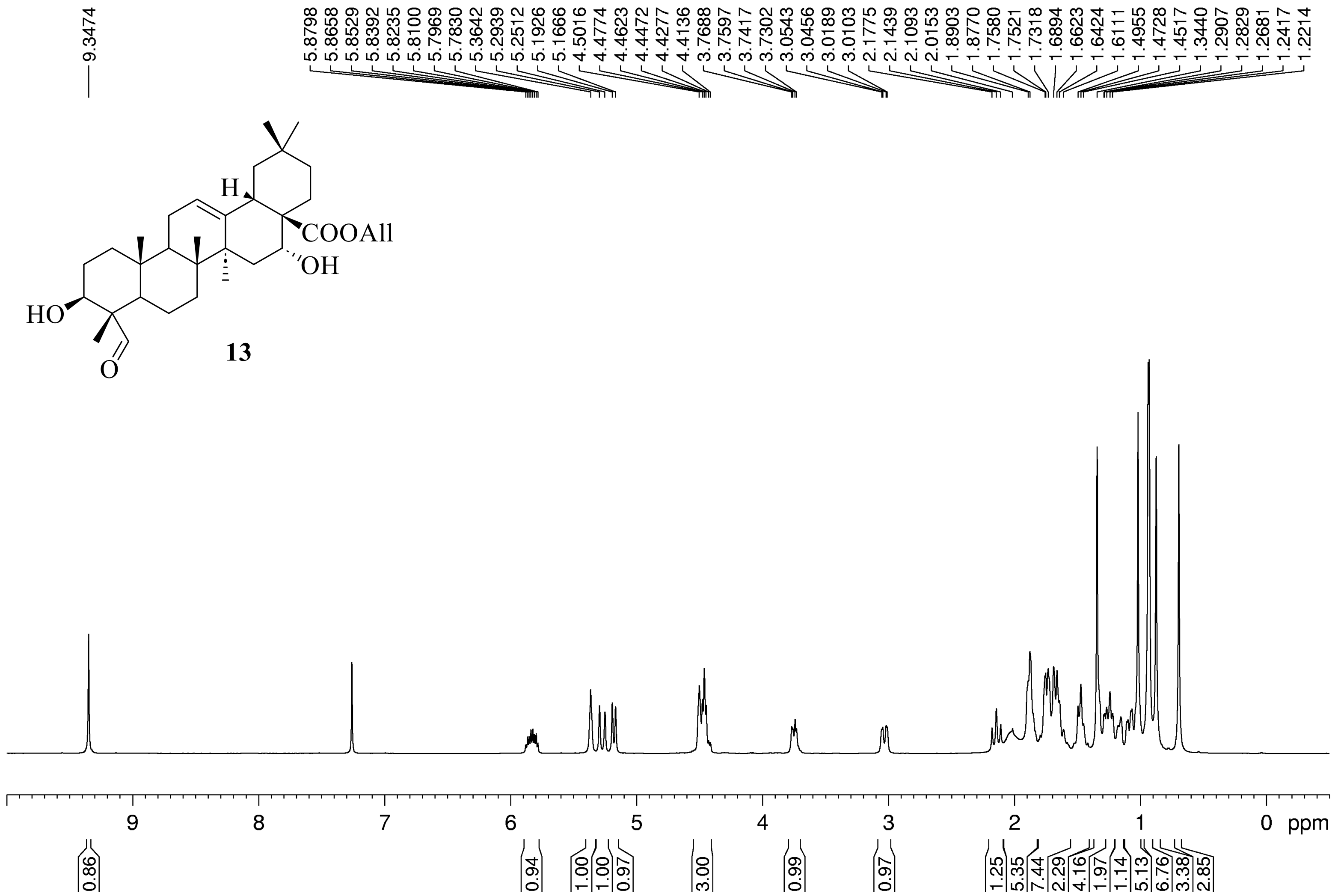

^{1}H NMR spectrum of **13** ($CDCl_3$, 600 MHz)

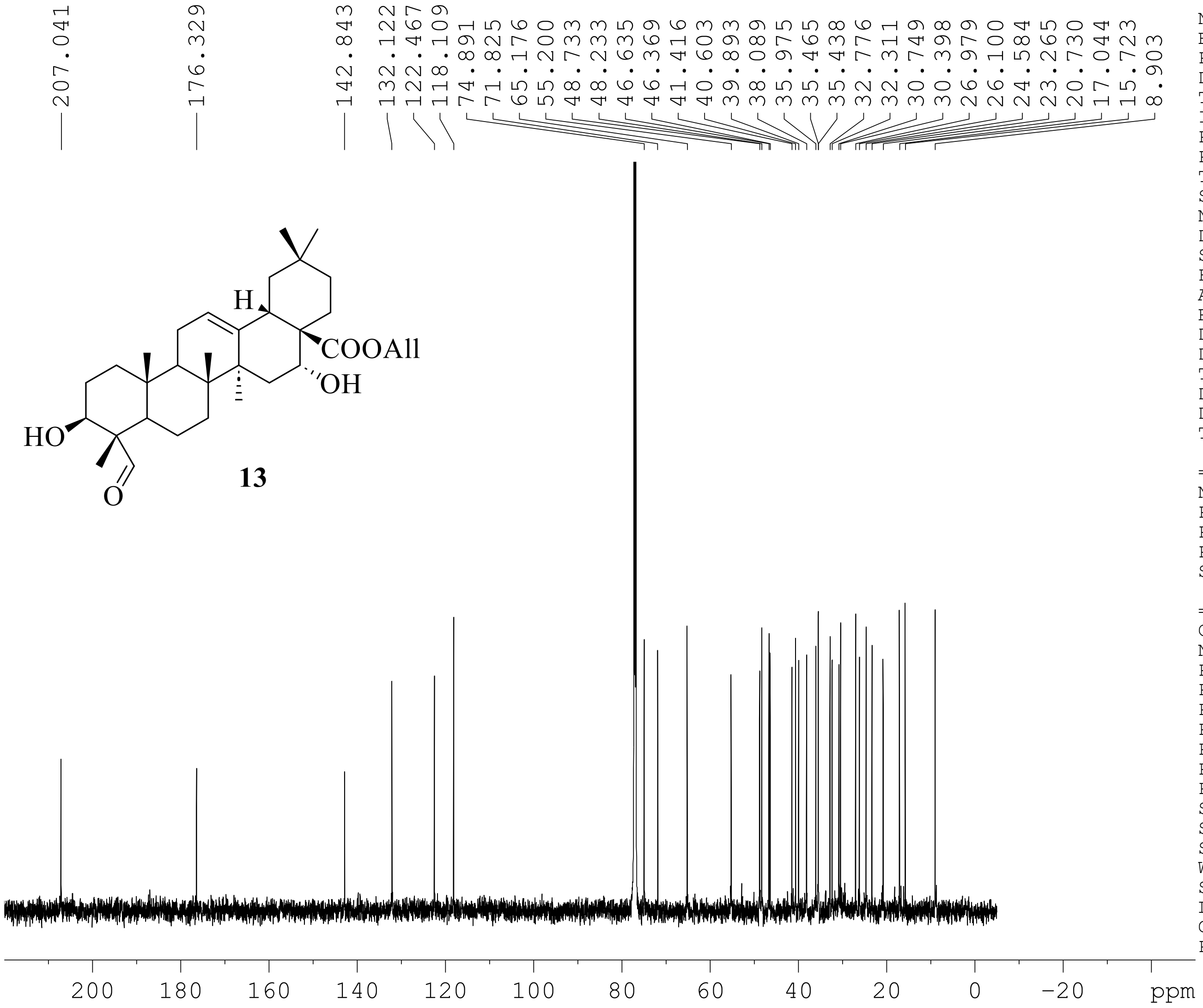

NAME phl-ccr-20221214-cpd1
EXPNO 10
PROCNO 1
Date_ 20221214
Time 16.24
INSTRUM spect
PROBHD 5 mm PADUL 13C
PULPROG zgpg30
TD 65536
SOLVENT CDCl3
NS 1024
DS 0
SWH 34722.223 Hz
FIDRES 0.529819 Hz
AQ 0.9437684 sec
RG 2050
DW 14.400 usec
DE 20.00 usec
TE 297.5 K
D1 2.00000000 sec
D11 0.03000000 sec
TD0 1

======== CHANNEL f1 ========
NUC1 13C
P1 10.00 usec
PL1 4.00 dB
PL1W 36.50000000 W
SFO1 150.9194069 MHz

======== CHANNEL f2 ========
CPDPRG2 waltz16
NUC2 1H
PCPD2 90.00 usec
PL2 0.20 dB
PL12 16.00 dB
PL13 19.00 dB
PL2W 15.37651920 W
PL12W 0.40444365 W
PL13W 0.20270200 W
SFO2 600.1324005 MHz
SI 32768
SF 150.9028135 MHz
WDW EM
SSB 0
LB 3.00 Hz
GB 0
PC 1.00

$^{13}C\{^{1}H\}$ NMR spectrum of **13** ($CDCl_3$, 151 MHz)

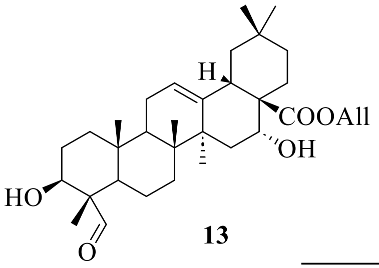

13

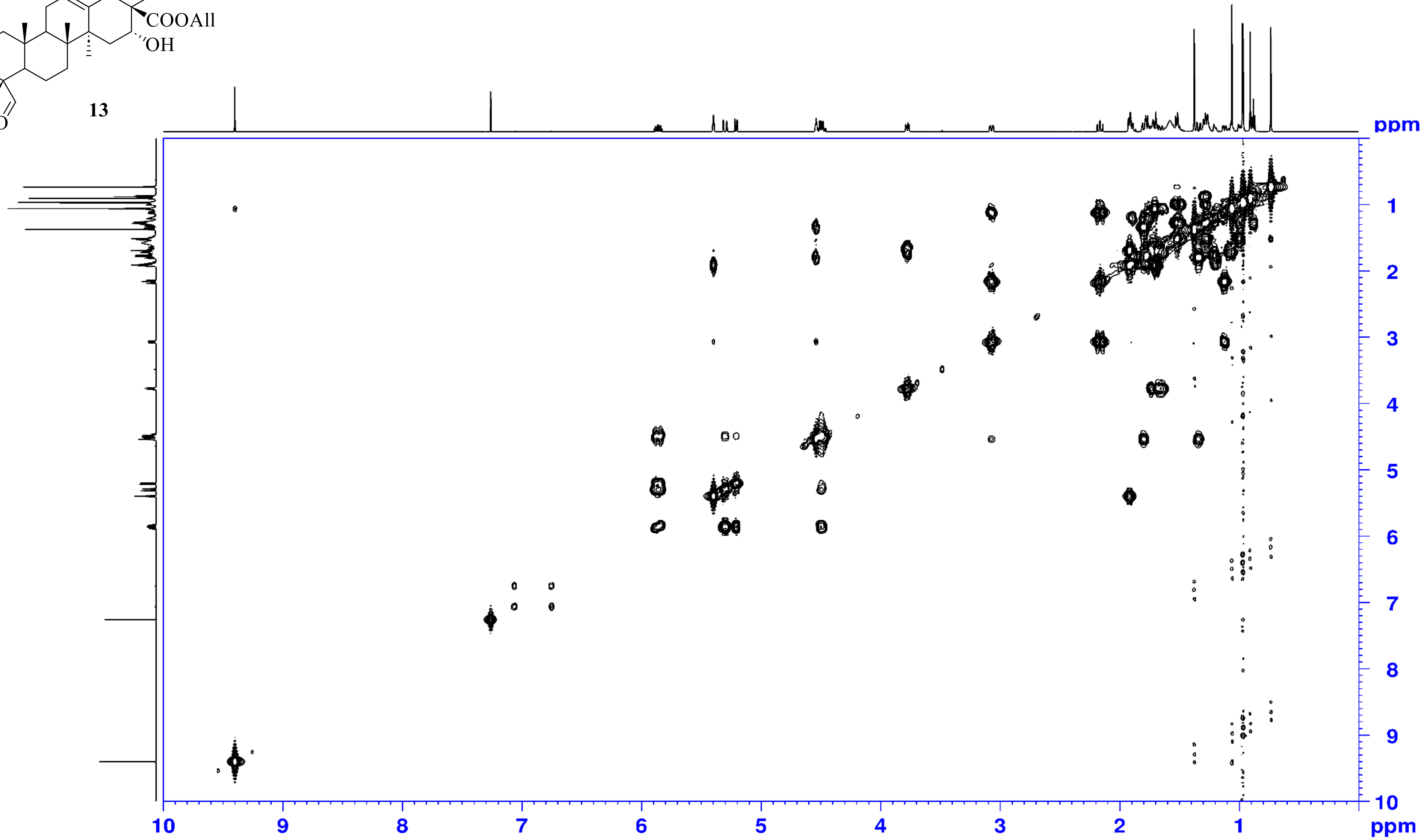

COSY of **13** ($CDCl_3$, 600 MHz)

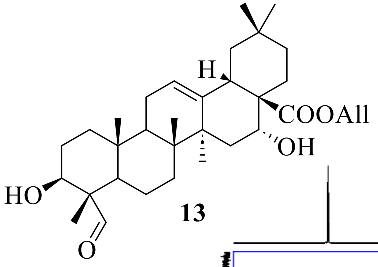

13

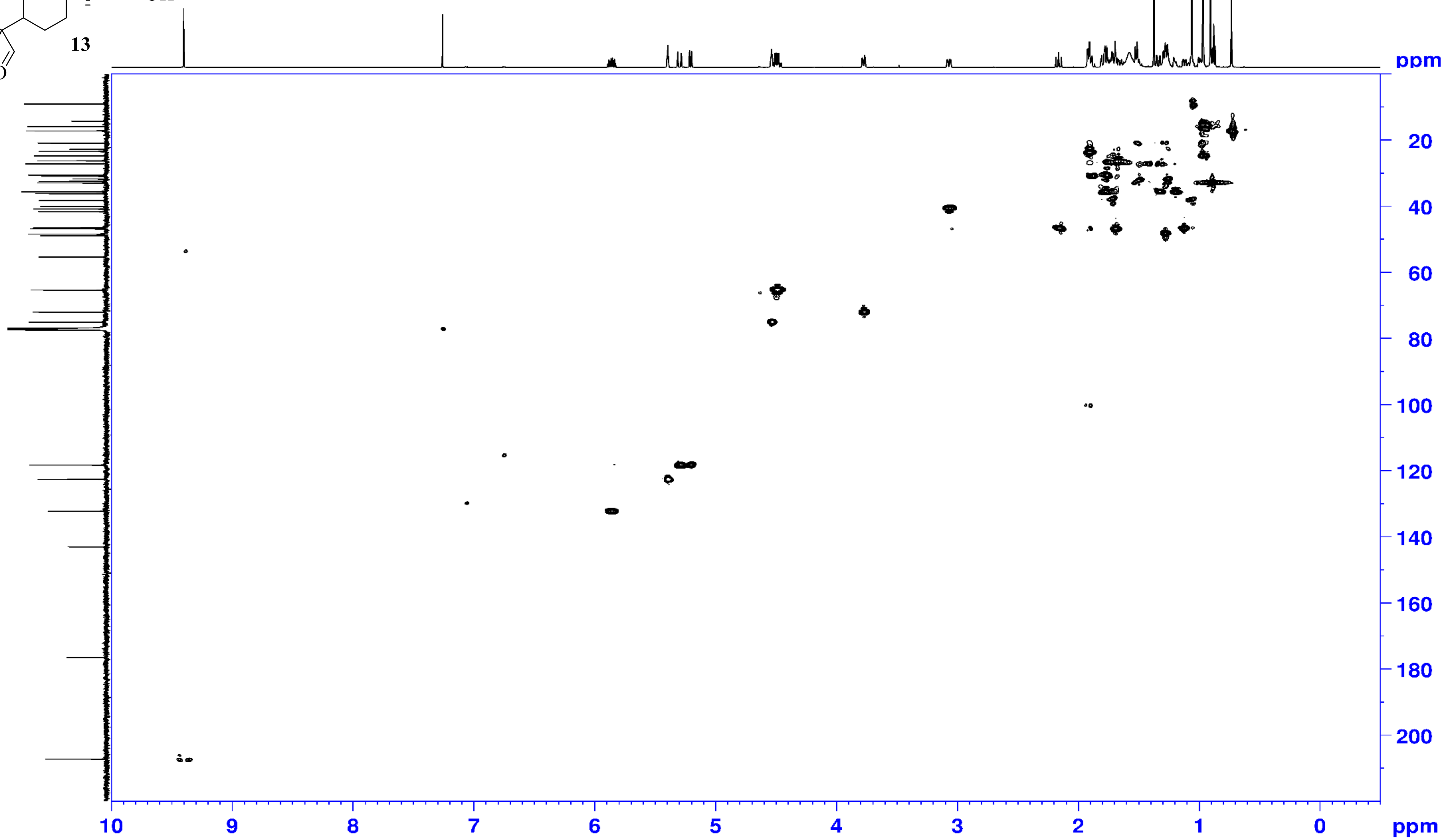

HSQC of **13** ($CDCl_3$, 600 MHz)

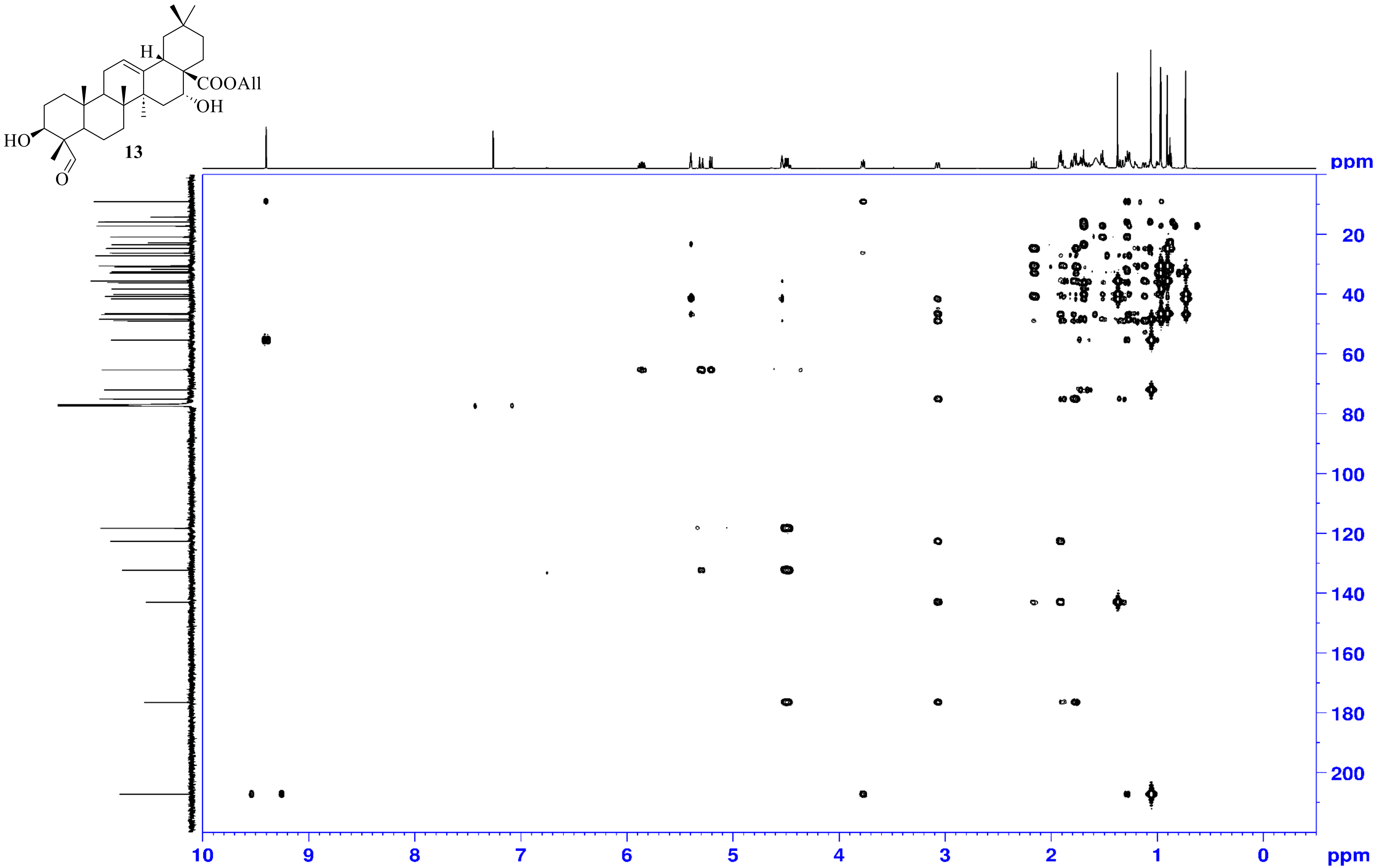

HMBC of **13** ($CDCl_3$, 600 MHz)

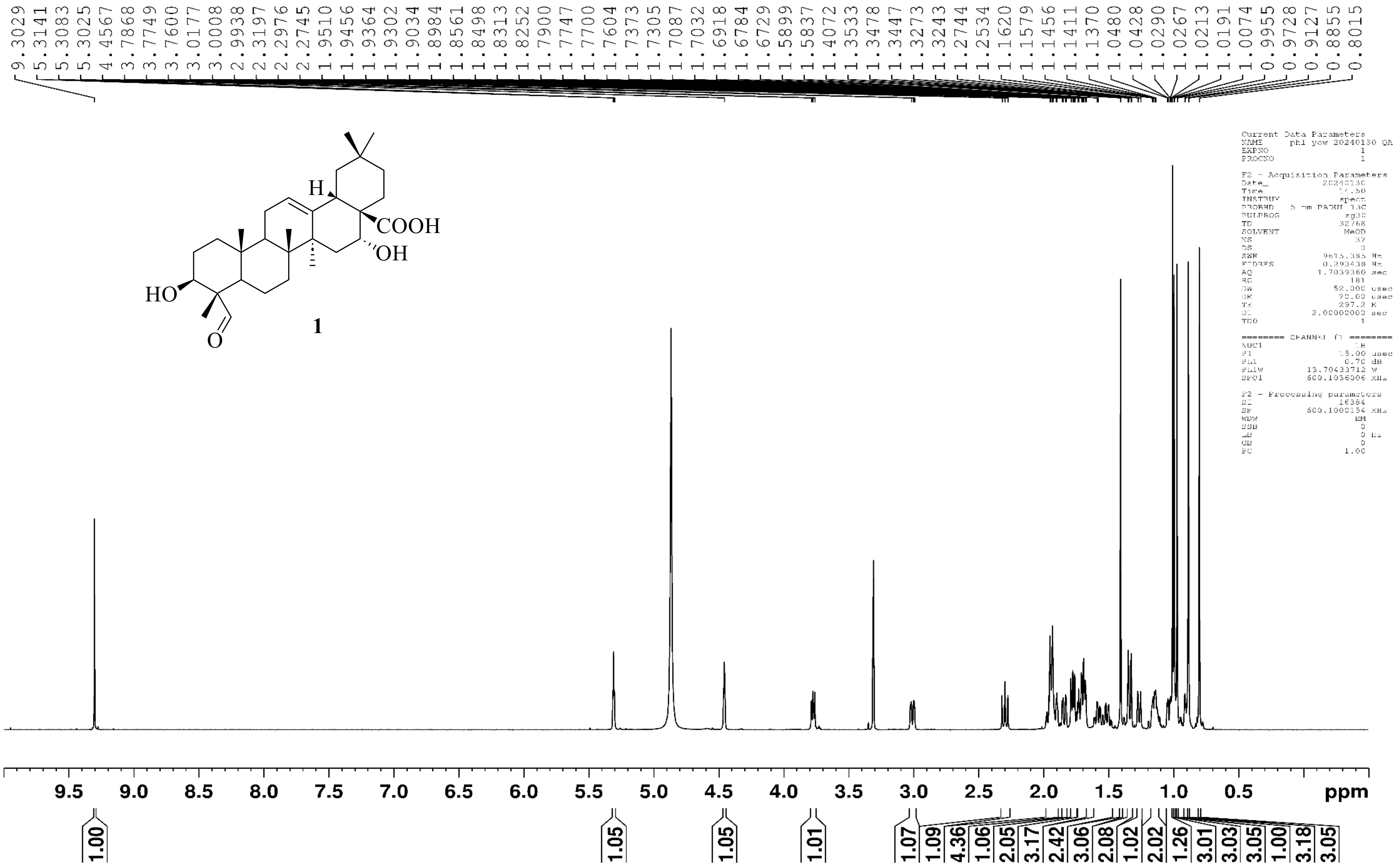

^{1}H NMR spectrum of quillaic acid (**1**) (CD_3OD, 600 MHz)

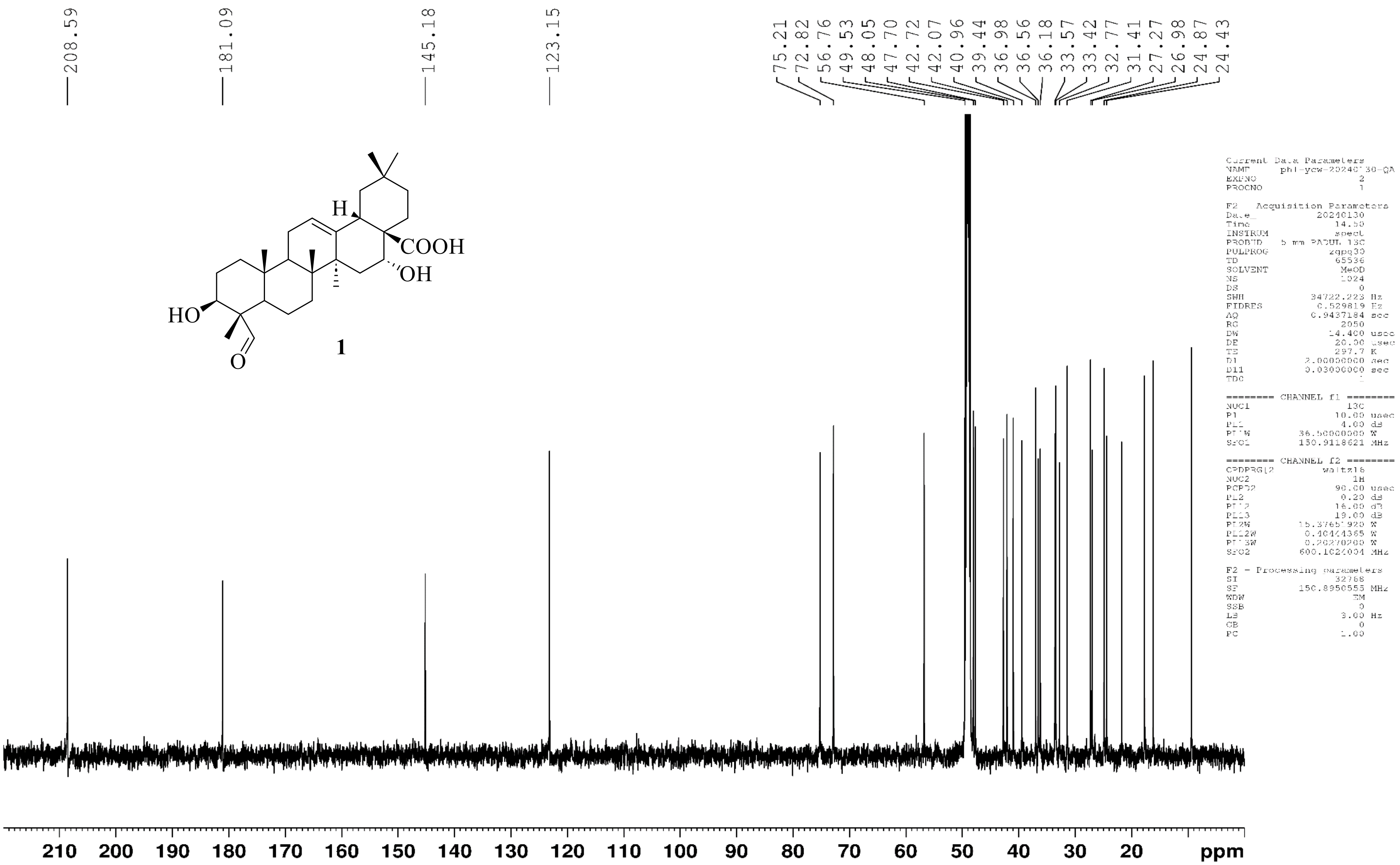

$^{13}C\{^{1}H\}$ NMR spectrum of quillaic acid (**1**) (CD_3OD, 151 MHz)

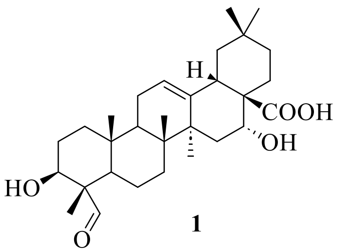

1

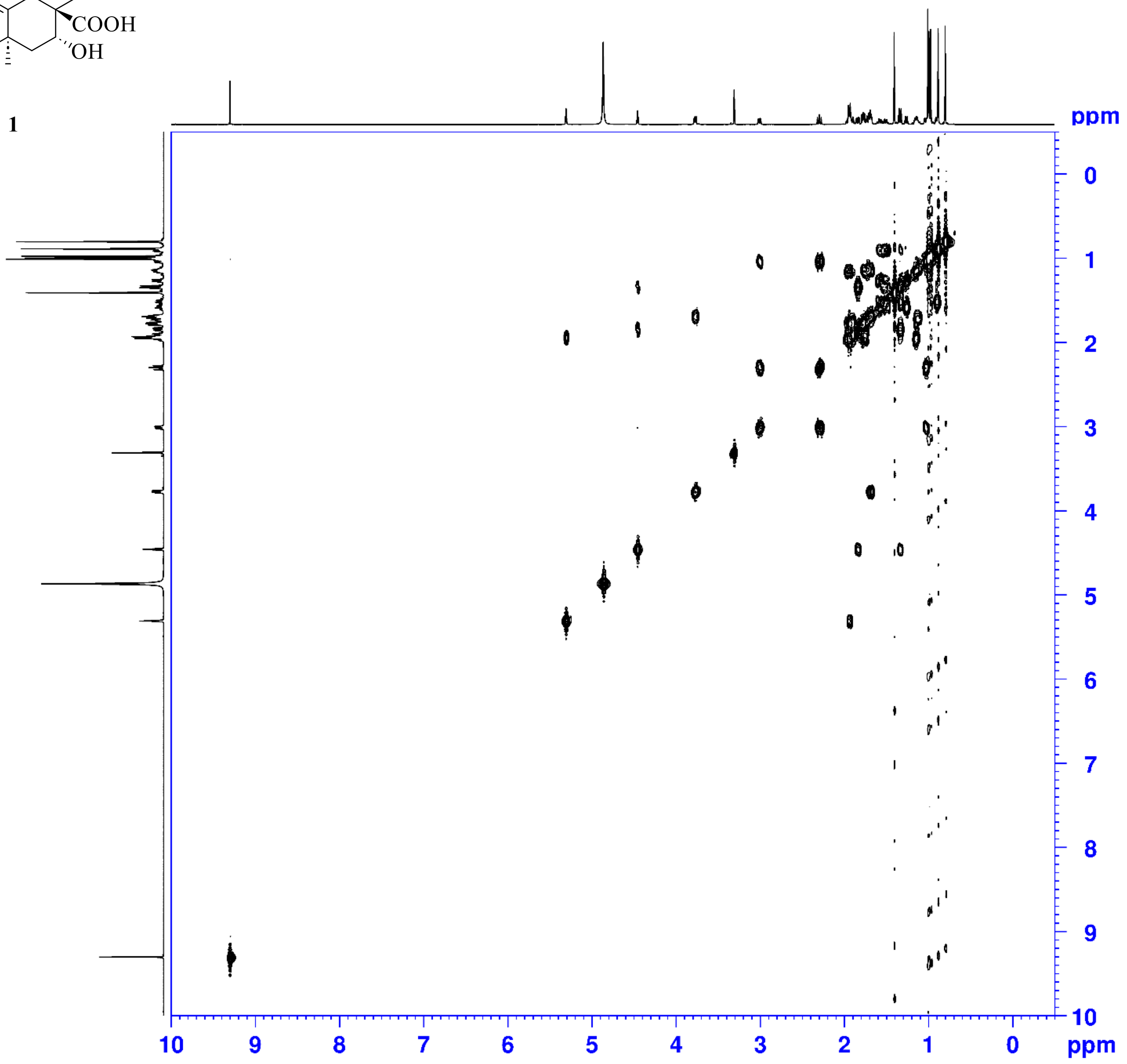


```
Current Data Parameters
NAME       phl-ycw-20240130-QA
EXPNO                        5
PROCNO                       1

F2 - Acquisition Parameters
Date_                 20240130
Time                     16.09
INSTRUM                  spect
PROBHD     5 mm PADUL 13C
PULPROG           cosygpqf45
TD                        2048
SOLVENT                   MeOD
NS                           4
DS                          16
SWH                   8012.820 Hz
FIDRES                3.912510 Hz
AQ                   0.1277952 sec
RG                         812
DW                      62.400 usec
DE                       20.00 usec
TE                       297.9 K
D0                  0.00000300 sec
D1                  2.00000000 sec
D13                 0.00000400 sec
D16                 0.00020000 sec
IN0                 0.00012480 sec

======== CHANNEL f1 ========
NUC1                        1H
P0                        7.50 usec
P1                       15.00 usec
PL1                       0.70 dB
PL1W               13.70433712 W
SFO1               600.1036006 MHz

====== GRADIENT CHANNEL =====
GPNAM[1]              SINE.100
GPZ1                     10.00 %
P16                    1000.00 usec

F1 - Acquisition parameters
TD                         256
SFO1                  600.1036 MHz
FIDRES               62.590252 Hz
SW                      13.350 ppm
FnMODE                      QF

F2 - Processing parameters
SI                        1024
SF                 600.1000180 MHz
WDW                       SINE
SSB                          0
LB                           0 Hz
GB                           0
PC                        1.40

F1 - Processing parameters
SI                        1024
MC2                         QF
SF                 600.1000100 MHz
WDW                       SINE
SSB                          0
LB                           0 Hz
GB                           0
```

COSY of quillaic acid (**1**) (CD_3OD, 600 MHz)

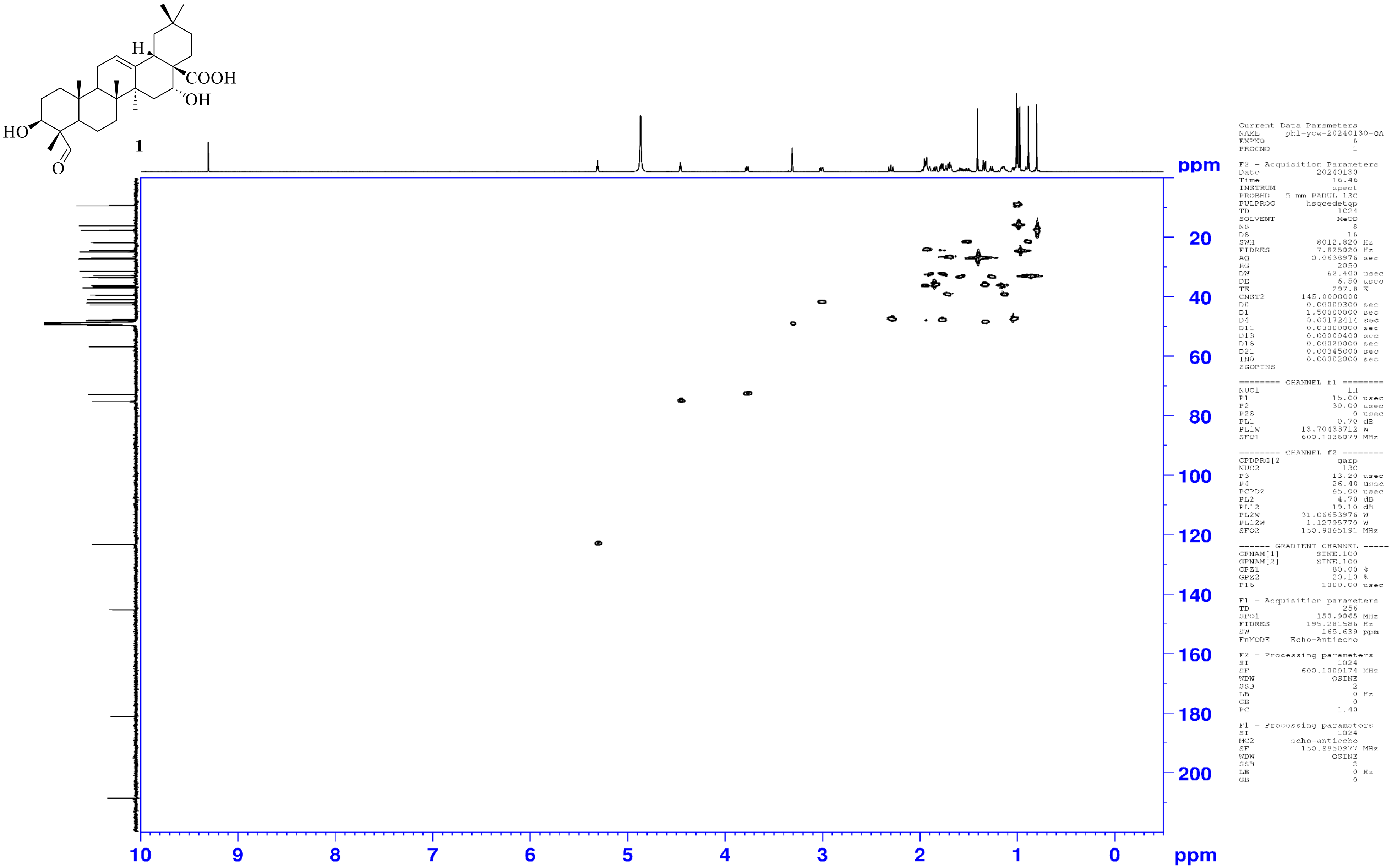

HSQC of quillaic acid (**1**) (CD_3OD, 600 MHz)

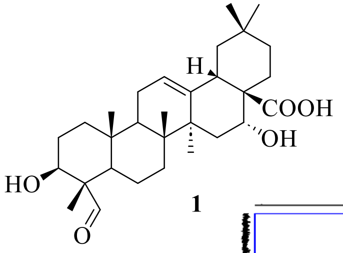

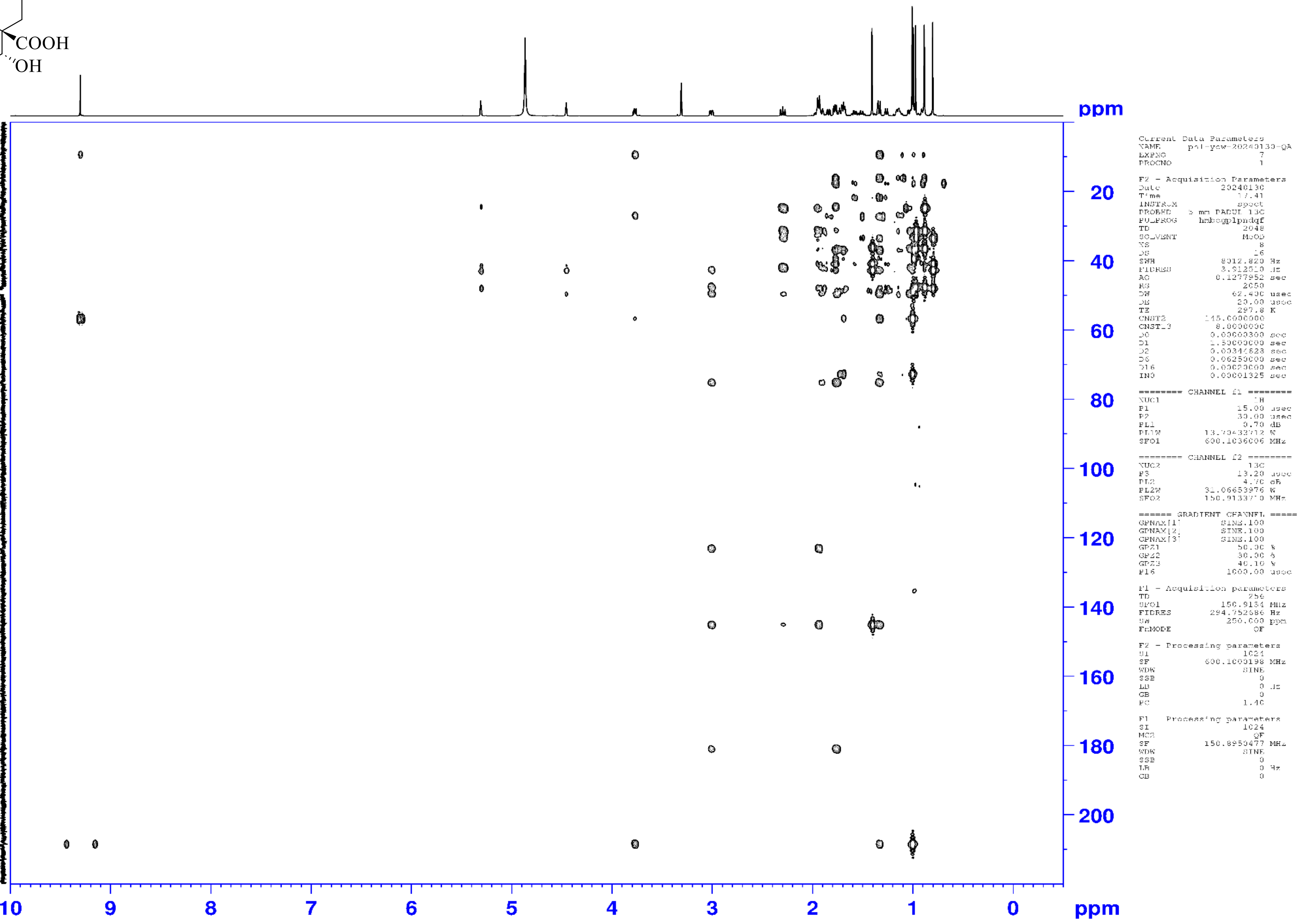

HMBC of quillaic acid (**1**) (CD$_3$OD, 600 MHz)
